# Embryonic organizer formation disorder leads to multiorgan dysplasia in Down syndrome

**DOI:** 10.1038/s41419-022-05517-x

**Published:** 2022-12-19

**Authors:** Yanyan Liu, Ziyuan Lin, Ying Peng, Yan Jiang, Xuan Zhang, Hongmei Zhu, Lili Zhang, Jiurong Chen, Xianghua Shu, Min Luo, Dan Xie, Yan Chen, Huijuan Liao, Mingfeng Liu, Xiaohu Zhang, Shanling Liu, He Wang, Bin Zhou, Huaqin Sun

**Affiliations:** 1 Prenatal Diagnosis Center, Department of Medical Genetics, Department of Obstetrics & Gynecologic, Key Laboratory of Birth Defects and Related Diseases of Women and Children (Sichuan University), Ministry of Education, West China Second University Hospital, Sichuan University, Chengdu, 610041 People’s Republic of China; 2 SCU-CUHK Joint Laboratory for Reproductive Medicine, Key Laboratory of Birth Defects and Related Diseases of Women and Children (Sichuan University), Ministry of Education, Department of Pediatrics, West China Second University Hospital, Sichuan University, Chengdu, 610041 People’s Republic of China; 3 Department of Cardiology, West China Hospital, Sichuan University, Chengdu, Sichuan Province 610041 People’s Republic of China; 4 Department of prenatal Diagnosis, Mianyang People’s Hospital, Mianyang, 621000 Sichuan Province People’s Republic of China; 5 Department of Obstetrics, Mianyang People’s Hospital, Mianyang, 621000 Sichuan Province People’s Republic of China; 6 Laboratory of Molecular Translational Medicine, Center for Translational Medicine, Department of Obstetrics and Gynecology, Key Laboratory of Birth Defects and Related Diseases of Women and Children (Sichuan University), Ministry of Education, West China Second University Hospital, Sichuan University, Chengdu, 610041 People’s Republic of China

**Keywords:** Disease model, Embryogenesis, Experimental models of disease

## Abstract

Despite the high prevalence of Down syndrome (DS) and early identification of the cause (trisomy 21), its molecular pathogenesis has been poorly understood and specific treatments have consequently been practically unavailable. A number of medical conditions throughout the body associated with DS have prompted us to investigate its molecular etiology from the viewpoint of the embryonic organizer, which can steer the development of surrounding cells into specific organs and tissues. We established a DS zebrafish model by overexpressing the human *DYRK1A* gene, a highly haploinsufficient gene located at the “critical region” within 21q22. We found that both embryonic organizer and body axis were significantly impaired during early embryogenesis, producing abnormalities of the nervous, heart, visceral, and blood systems, similar to those observed with DS. Quantitative phosphoproteome analysis and related assays demonstrated that the DYRK1A-overexpressed zebrafish embryos had anomalous phosphorylation of β-catenin and Hsp90ab1, resulting in Wnt signaling enhancement and TGF-β inhibition. We found an uncovered ectopic molecular mechanism present in amniocytes from fetuses diagnosed with DS and isolated hematopoietic stem cells (HSCs) of DS patients. Importantly, the abnormal proliferation of DS HSCs could be recovered by switching the balance between Wnt and TGF-β signaling in vitro. Our findings provide a novel molecular pathogenic mechanism in which ectopic Wnt and TGF-β lead to DS physical dysplasia, suggesting potential targeted therapies for DS.

## Introduction

Down syndrome (DS), which affects 1 in 600–800 babies born in all populations, is the most frequently occurring chromosomal abnormality in humans [1]. A number of medical conditions are associated with DS. Intellectual disability, cardiac defects, leukemia, gastrointestinal issues, vision and hearing issues, dental issues, thyroid disease, obstructive sleep apnea, epilepsy, and Alzheimer’s disease are the most common conditions. The physical features of infants with DS at birth are distinctive, including short neck, small ears, flat nasal bridge, epicanthal folds, brushfield spots, single palmar crease, small mouth with large protruding tongue, cardiac murmur, hypotonia, poor respiratory effort, poor sucking, abdominal distension, and vomiting [2]. Congenital cardiac defects are observed in half of the babies with DS, involving the ventricular septum, tetralogy of Fallot, and the atrioventricular canal [3]. Congenital gastrointestinal anomalies also exist in 12% of babies with DS, including gastroesophageal reflux disease, coeliac disease tracheoesophageal fistula, Meckel diverticulum, esophageal or duodenal atresia, imperforate anus, pyloric stenosis, and Hirschsprung disease [4].

Organizers, defined as groups of cells with the ability to instruct adjacent cells to differentiate into specific states, represent a key principle in developmental biology. In the context of an embryo, they are groups of cells with the ability to direct fates and morphogenesis in surrounding cells, steering their development into specific organs and tissues. As a result, organizers can position specific tissues and organs relative to each other [5]. Therefore, the additional health issues associated with DS inspired us to investigate its etiology from the viewpoint of embryonic organizers.

The gene-dosage hypothesis is the most accepted theory for the pathogenesis of DS; that is, the syndrome is caused by chromosome 21 trisomy in somatic cells, causing increased expression of chromosome 21 genes due to gene dosage [1, 6, 7]. Transgenic mouse data suggest that only some genes on chromosome 21, particularly those in the “critical region” at 21q22, may be involved in the phenotypes of DS, with some gene products more sensitive to gene dosage imbalance than others [8, 9]. In addition, when sensitivity to haploinsufficiency (i.e., intolerability to heterozygous loss-of-function alleles) is used to rank human chromosome 21 (HSA21) protein-coding genes, several high-ranking genes, such as *DYRK1A*, have already been associated with specific clinical features of DS. It has been argued that human chromosome 21 (HSA21) protein-coding genes with high haploinsufficiency scores are more sensitive to three copies; therefore, they can be considered candidates for contributing to DS phenotypes [10].

Indeed, several studies have shown that the overexpression of *DYRK1A*, in 21q22, contributes to DS phenotypes [10–12]. Overexpression of *Dyrk1A* in mice, mBACTgDyrk1A (*Dyrk1A*^+/++^), is sufficient to produce significant behavioral impairments, cognitive deficits, motor abnormalities, neuronal alterations, retinal defects, vascular defects, gastrointestinal tract abnormalities, immune response defects, abnormal maturation of the thymus, and impaired function of T lymphocytes [13–15] that recapitulate those detected in human trisomy 21 [16–18]. Zebrafish biology allows ready access to all developmental stages, and the optical clarity of embryos and larvae allows real-time imaging of developing pathologies [19]. Based on these data and the mouse model described above, we established a DS zebrafish model by overexpressing the human *DYRK1A* gene.

The zebrafish embryonic organizer was disrupted by *DYRK1A* overexpression, and the body axis, associated with multi-organ disorders in early development, was also disrupted. Our molecular characterization of this model demonstrated that Wnt and TGF-β signaling both changed significantly. We verified this mechanism in amniocytes, and isolated hematopoietic stem cells (HSCs) from fetuses and patients diagnosed with DS. We also tested switching Wnt and TGF-β signaling simultaneously to rescue these abnormalities in zebrafish embryos and DS patient HSCs, providing potential guidance for DS therapies. We also provide a new molecular mechanism for embryonic organizer formation and body axis development.

## Results

### *DYRK1A* overexpression in zebrafish disrupts the embryonic organizer and body axis

**Fig. 1:**
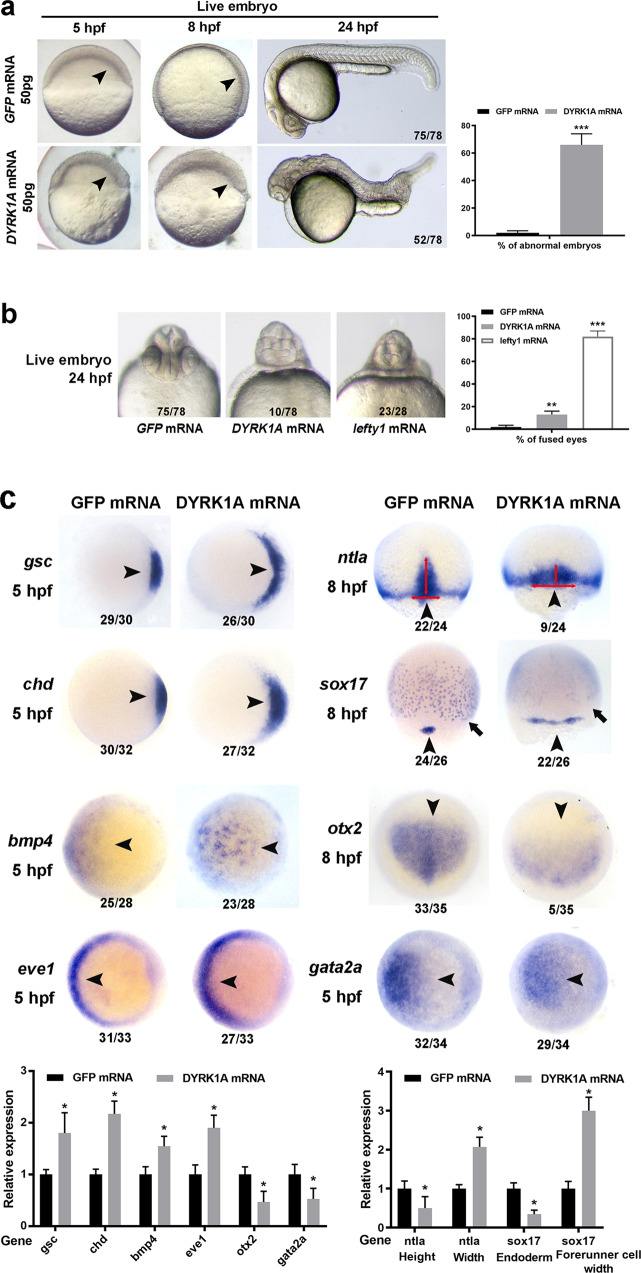
DYRK1A-overexpressed zebrafish embryo model showed shortened/curved body axis. **a** Morphogenesis of live control (injection with GFP mRNA) and DYRK1A-overexpressed (injection with DYRK1A mRNA) embryos. The arrowheads indicate the dorsal side of the embryo. **b** Fusion of eyes caused by DYRK1A overexpression resembled the phenotype of lefty1 mRNA injection. **c** Germ layer marker expression detected by WISH during gastrulation. Orientation: ntla, sox17, and otx2, dorsal views with an animal pole to the top; others, animal-pole views with dorsal to the right. The numbers indicated in each picture are the number (left) of affected embryos with a phenotype similar to what is shown in the picture and the total number (right) of observed embryos. The same number labeling was used thereafter.

We injected human *DYRK1A* or zebrafish *dyrk1a* mRNA into zebrafish embryos. When higher amounts of mRNA (~100 pg) were injected, *DYRK1A* mRNAs produced frequent embryo death at 24 h post fertilization (hpf) (Extended Data Fig. 1). However, when we decreased the mRNA level to ~50 pg, we observed a lower rate of embryo death with severe embryonic abnormalities in body axis development, including shortened/curved axis and fusion of the eyes (Fig. 1a, b).

We next constructed a transgenic zebrafish line using the Gal4-UAS system to overexpress *DYRK1A*. We established two Gal4 lines: gsc:Gal4 and β-actin; and Gal4, to overexpress *DYRK1A* from a UAS element in specific organizer cells or ubiquitously in embryos. Similar to the phenotype obtained from *DYRK1A* mRNA injection, both Tg (gsc:Gal4; UAS:*DYRK1A*) and Tg (β-actin:Gal4; UAS:*DYRK1A*) embryos exhibited shortened/curved body axes and fusion of the eyes (Extended Data Fig. 2).

We next assessed the tissue defects in our DS embryonic model. We found that the system of nerve, heart, blood, and germ cells [20] in the embryos exhibited significant dysplasia (Extended Data Figs. 3 and 4). At 24 hpf, we observed a significant decrease in the expression levels of the nervous stem cell marker *neurog* and the hindbrain neuronal marker *pax2a*. The neural plate, marked by *the sox3* gene, was disordered in the DS embryonic model (Extended Data Fig. 3a). At 48 hpf, DYRK1A-overexpressed embryos had defective heart tubes located at the midline and to the right (marked by *myl7*), while the expression level of the atrioventricular boundary markers *bmp4* and *has2* was decreased in the DYRK1A-overexpressed embryos (Extended Data Fig. 3b). At 72 hpf, the gut marker gene *fabp10a* also showed reduced expression (Extended Data Fig. 3c). Furthermore, the DYRK1A-overexpressed embryos demonstrated severe hematopoietic abnormalities. Expression levels of the hematopoietic stem-cell marker genes *runx1* (28 hpf) and *cmyb* (48 hpf), as well as those of the hematopoietic progenitor cell marker genes *scl* and *gata1* (13 hpf), were significantly increased in model embryos (Extended Data Fig. 4a, c). However, the T cell marker genes *rag1* and *rag2* (4 dpf), the macrophage marker gene *csf1ra*, and the primitive neutrophil marker gene *mpx* (24 hpf) showed decreased expression (Extended Data Fig. 1b and S4d). Additionally, we did not detect changes in the expression of blood vessel marker genes (Extended Data Fig. 4e). These multi-organ disorders were consistent with those observed in patients with DS.

**Fig. 2:**
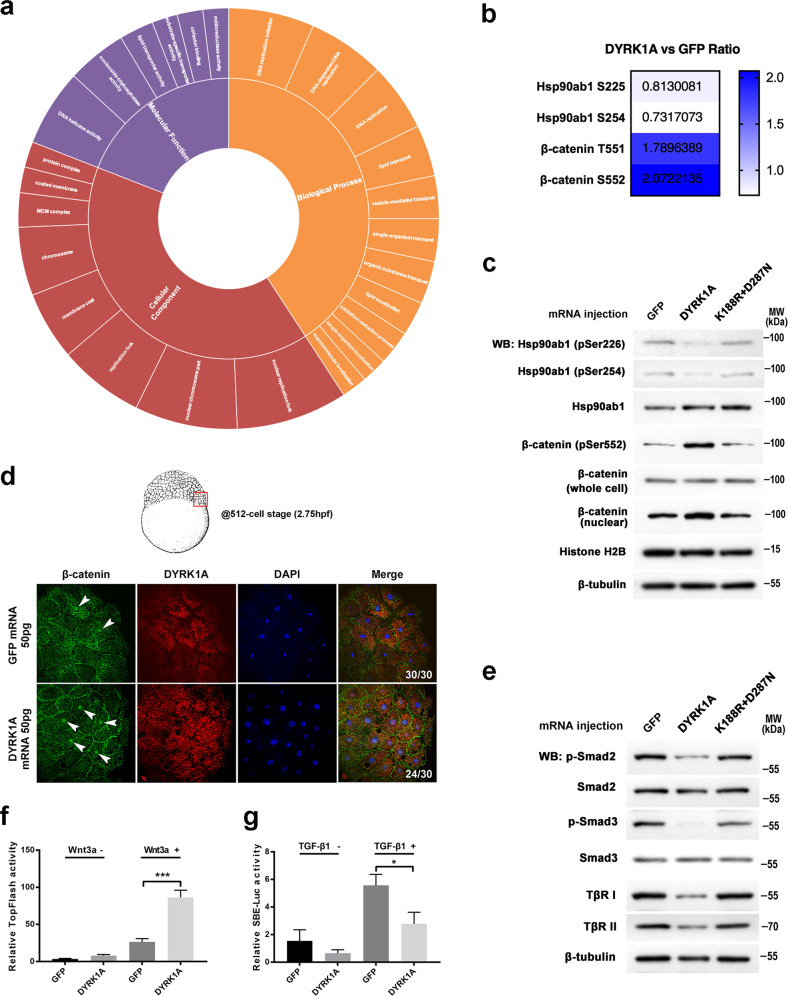
Quantitative phosphoproteome analysis shows the altered phosphorylation level essential for organizer and body axis development in DYRK1A-overexpressed embryos. **a** Gene Ontology (GO)-based enrichment analysis of regulated protein phosphorylation sites on the ontology of biological processes, cellular components, and molecular functions. **b** The quantified phosphorylation sites of β-catenin and Hsp90ab1 were detected by phosphoproteome analysis in DYRK1A-overexpressed embryos. **c** Regulation of phosphorylation sites of β-catenin and Hsp90ab1 was verified using western blot assay in zebrafish embryos. **d** Whole-mount immunostaining of β-catenin at the 512-cell stage showed that nuclear β-catenin increased in the DYRK1A-overexpressed embryo. Whole embryos were flattened and viewed from the animal pole by confocal microscopy. **e** DS embryo model blocks Smad2/3 phosphorylation detected by western blot. **f** DYRK1A enhanced the expression of the Wnt reporter TopFlash in HEK-293T cells. (**g**) DYRK1A decreased TGF-β induced SBE-luc activity in HEK-293T cells.

In our previous study, we showed that zebrafish *dyrk1a* mRNA and protein were expressed in all blastodermal cells and the axis ubiquitously during early embryogenesis, suggesting a potential role of *dyrk1a* in body axis development. Interestingly, zebrafish *dyrk1a* mRNA was concentrated on the dorsal organizer side at the shield stage (Fig. 2 and Supplemental Fig. S1 in Liu’s paper) [20], suggesting a potential role of *dyrk1a* in organizer formation. Therefore, these results also give us a reason to investigate DS etiology from the perspective of the embryonic organizer and body axis.

Next, we quantified the expression of several marker genes related to the organizer and germ layer body axis. By whole-mount in situ hybridization (WISH), expression levels of the dorsal organizer markers *gsc* and *chd* were found to be increased significantly in DYRK1A-overexpressed embryos at the shield stage. The expression levels of the ventral markers *bmp4* and *eve1* also increased. Consistent with *gsc* and *chd* expression, the axial mesoderm, marked by *ntla* expression, became wider and shorter. The axial forerunner cell population marked by *sox17* also showed a wider pattern. Expression of the endodermal marker *sox17*, the anterior neuroectoderm marker *otx2*, and the epidermis marker *gata2a* all decreased during the gastrula period (Fig. 1c). These data suggest that our established DS embryonic model by overexpression of DS “critical region” gene *DYRK1A* induce developmental abnormalities of the body axis, including organizer and three germ layers, corresponding a number of medical conditions associated with DS.

To determine whether the increased expression of organizer markers *gsc* and *chd* in DYRK1A-overexpressed was a consequence of the increased proliferation of organizer cells, we analyzed organizer cells using a phospho-histone H3 (pH3) antibody and counterstaining with GFP in a *Tg(gsc:GFP)* transgenic fish line. We observed a significant increase in the number of proliferating cells in the organizer (Extended Data Fig. 2), suggesting that the DYRK1A-overexpressed embryos induced aberrant cell proliferation of embryonic organizer cells.

Taken together, these data suggest that in the DS embryo model, there is the ectopic proliferation of organizer cells and mesoderm that led to decreased differentiation of ectoderm and endoderm.

### Aberrant phosphorylation of key factors in the establishment of the organizer and body axis in DYRK1A-overexpressed embryos

Since DYRK1A (dual-specificity tyrosine phosphorylation-regulated kinase 1A) is a protein kinase [21], we used TMT labeling and phosphorylation affinity enrichment followed by high-resolution LC-MS/MS to quantify the changes in the whole phosphoproteome of zebrafish embryos at the 50% epiboly stage in embryos injected with human *DYRK1A* and GFP mRNA (see Fig. 5a in reference [20] for the procedure). Increased DYRK1A protein expression levels in embryos injected with *DYRK1A* mRNA have been shown previously [20]. Despite the high proportion of yolk proteins in early embryos, 951 phosphorylation sites in 553 protein groups were identified, of which 226 sites in 115 proteins were quantified in response to *DYRK1A* and GFP mRNA injection (Supplementary Table 1). The fold-change cutoff was set so that proteins with quantitative ratios above 1.2 or below 0.83 were deemed significant. Of the quantified proteins, 86 phosphorylation sites in 52 were hyperphosphorylated and 46 phosphorylation sites in 33 proteins were hypophosphorylated when compared to the control (Supplementary Table 2).

To characterize the function of these alterations in protein phosphorylation, Gene Ontology (GO)-based classification of the ontology of cellular components, molecular functions, and biological processes was performed, which revealed widely different distributions between DYRK1A-overexpressed and control embryos (Supplementary Table 3). The proteins with altered expression in the DYRK1A-overexpressed embryos were enriched for MCM complex, nuclear chromosome part, replication fork, chromosome, protein complex, nuclear replication fork, coated membrane, and membrane coat. Molecular function-based enrichment analysis identified oxidoreductase activity, DNA helicase activity, cofactor binding, lipid transporter activity, substrate-specific transporter activity, and nucleoside-triphosphatase activity in regulated proteins. For biological processes, oxidation-reduction, DNA replication initiation, DNA-dependent DNA replication, lipid transport, lipid localization, DNA replication, macromolecule localization, single-organism localization, organic substance transport, single-organism transport, vesicle-mediated transport were enriched in the regulated proteins (Fig. 2a).

**Fig. 3:**
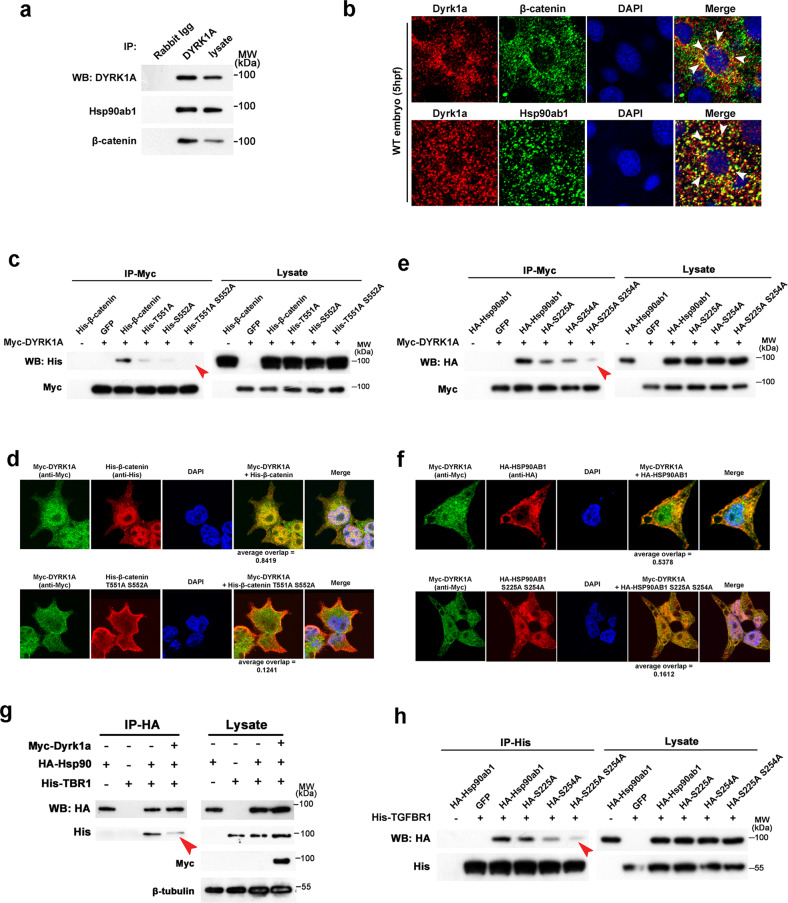
Phosphorylation sites of β-catenin and Hsp90ab1 are required for its interaction with DYRK1A and signaling transduction. **a** Co-immunoprecipitation (Co-IP) of endogenous DYRK1A and β-catenin/Hsp90ab1 in zebrafish embryos. **b** Co-localization of DYRK1A and β-catenin/Hsp90ab1 in the zebrafish embryo. Arrowheads indicate colocalized sites. **c** Co-IP shows the interaction of DYRK1A with β-catenin is abolished by T551A and S552A mutation. **d** Immunofluorescence analysis in HEK293 cells shows the reduced localization of β-catenin T551A and S552A mutant with DYRK1A. **e** Co-IP shows the interaction of DYRK1A with Hsp90ab1 is impaired by S225A and S254A mutation. **f** Immunofluorescence analysis in HEK293 cells shows that Hsp90ab1 S225A and S254A mutant is less co-localized with DYRK1A. **g** Co-IP/western blot analysis in HEK293 cells shows that DYRK1A overexpression decreases the protein–protein interaction between Hsp90ab1 and TGF-β receptor. **h** Hsp90ab1 S225 and S254 sites are required for its interaction with the TGF-β receptor. Red arrowheads in (**c**, **e**, **g**, **h**) indicate the decreased interaction of proteins.

**Fig. 4:**
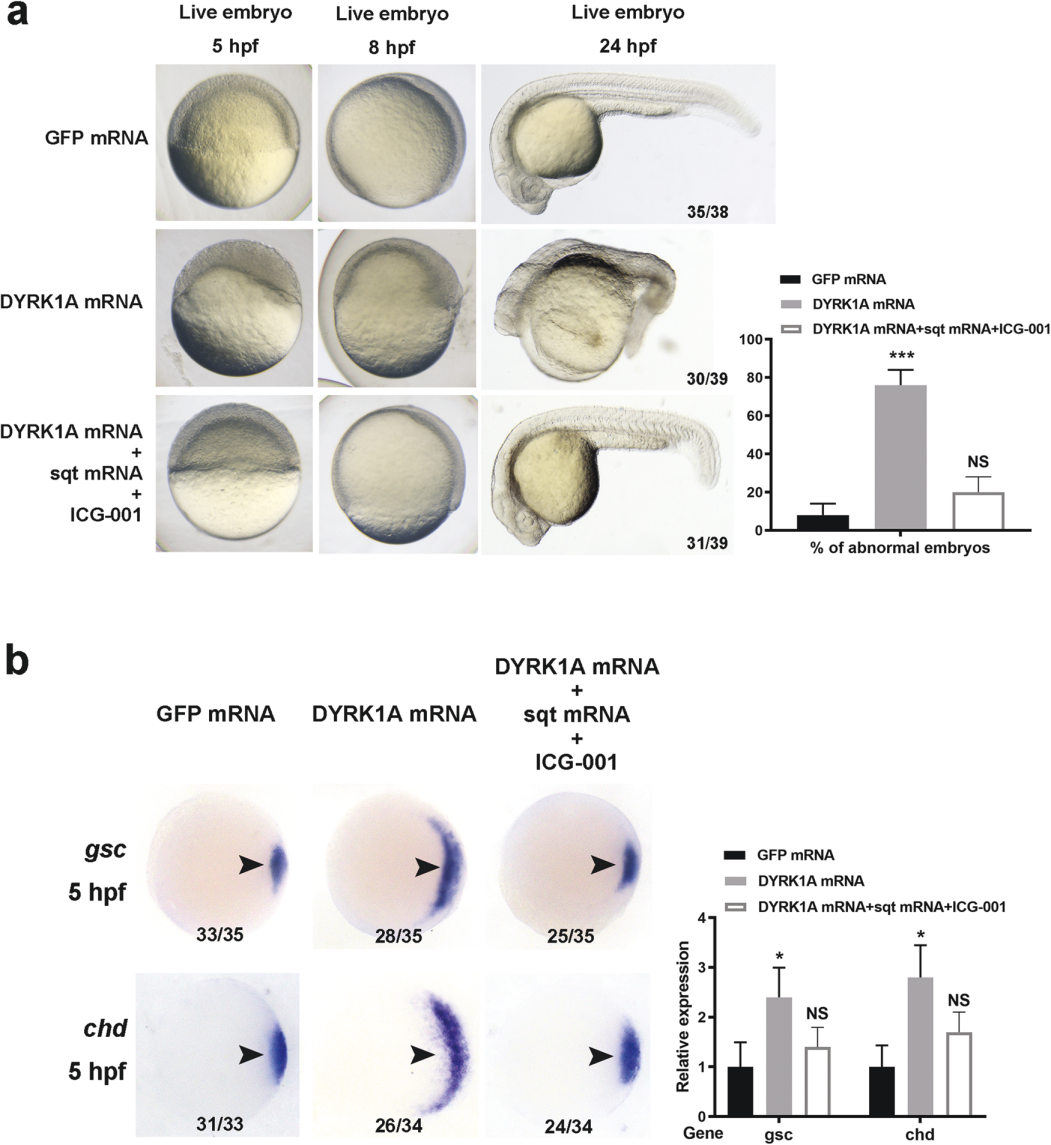
Regulation of Wnt and TGF-β signaling rescues embryo development and organizer formation in DYRK1A-overexpressed embryos. **a** Injection of TGF-β legend *sqt* mRNA and treatment of Wnt-β-catenin inhibitor ICG-001 simultaneously rescue the abnormal phenotype of the DYRK1A-overexpressed embryo. **b** *sqt* mRNA and ICG-001 also recover the ectopic expression of organizer marker genes *gsc* and *chd* in the DYRK1A-overexpressed embryo.

Interestingly, we found that the phosphorylation levels at residues T551 and S552 of β-catenin were significantly increased by *DYRK1A* mRNA (Fig. 2b). β-Catenin, the key effector of canonical Wnt signaling in the future dorsal blastomeres, plays an essential role in the formation of the organizer and the anteroposterior axis [22]. On the contrary, overexpression of *DYRK1A* caused fusion of the eyes in embryos at 24 hpf, resembling the results of lefty1 mRNA injection (Fig. 1b and Extended Data Fig. 2), which were caused by insufficient Nodal signaling in the TGF-β pathway [23]. Therefore, focusing on the regulatory factors of Nodal signaling in our phosphoproteome data, we found that phosphorylation of S225 and S254 of Hsp90ab1 was decreased in *DYRK1A* mRNA injected embryos (Fig. 2b). Hsp90 is a molecular chaperone that facilitates the folding and stabilization of many protein kinases and intracellular signaling molecules. Inhibition of Hsp90 function blocks TGF-β-induced signaling and transcriptional responses. Furthermore, inhibition of Hsp90 function increases TGF-β receptor ubiquitination and degradation, which is dependent on the Smurf2 ubiquitin E3 ligase [24]. Of note, sites T551 and S552 of β-catenin and S225 and S254 of Hsp90ab1 are conserved between humans and zebrafish (Extended Data Fig. 6), suggesting the importance of these protein sites in vertebrates.

To validate the role of DYRK1A in regulating the phosphorylation of β-catenin and Hsp90ab1, we performed kinase assays in vivo and in vitro. In injected zebrafish embryos and transfected HEK293T cells, overexpression of DYRK1A altered the phosphorylation levels of β-catenin S552 and Hsp90ab1 S225/S254 residues. In contrast, the catalytically inactive double mutant (K188R and D287N) [25] failed to change phosphorylation at these three sites, as detected by phosphospecific antibodies that recognize β-catenin S552 and Hsp90ab1 S225/S254 sites (Fig. 2c, Extended Data Fig. 7a and Extended Data Fig. 8a).

Phosphorylation at Ser552 induces β-catenin accumulation in the nucleus and increases its transcriptional activity [26]. Interestingly, our IF experiments showed that β-catenin was present in the nuclei of fewer blastomere cells in GFP-injected control embryos at the 512-cell stage; however, the nuclear expression level of β-catenin increased in the DYRK1A-overexpressed embryo (Fig. 2d and Extended Data Fig. 7b), suggesting that the ectopic organizer cells were induced by increased β-catenin activity. Furthermore, we examined the effect of DYRK1A overexpression on Wnt3a-induced TopFlash luciferase activity in HEK293 cells and observed a significant enhancement (Fig. 2f), consistent with a role for DYRK1A in regulating Wnt signaling. Taken together, these results suggest an important role for DYRK1A in Wnt-dependent organizer formation and body axis development.

On the contrary, according to Wrighton et al. [24], we assessed the expression levels of Smad protein, Smad phosphorylation, and TGF-β receptor. Western blotting of whole-cell lysates revealed that DYRK1A overexpression significantly reduced TGF-β-induced phosphorylation of endogenous Smad2/3 (but not Smad2/3 protein expression) and the TGF-β receptor in both zebrafish embryos (Fig. 2e and Extended Data Fig. 7c) and HEK293 cells (Extended Data Fig. 8b). We also observed decreased nuclear accumulation of Smad2 upon TGF-β1 stimulation in DYRK1A-overexpressing HEK293 cells (Extended Data Fig. 9). We then evaluated the effect of DYRK1A overexpression on TGF-β responses in HEK293 cells using the synthetic TGF-β-responsive reporter gene SBE-luc, which is dependent on Smad activation. In control cells, TGF-β increased SBE-luc activity in HEK293 cells. However, the expression of *DYRK1A* significantly impaired TGF-β-induced responses (Fig. 2g). We also detected reduced expression of the TGF-β ligand *sqt* in DS zebrafish model embryos (Extended Data Fig. 10). In summary, DYRK1A overexpression decreased TGF-β signaling by dephosphorylating the Hsp90ab1 S225 and S254 residues.

### DYRK1A interacts with β-catenin and Hsp90ab1 in a phosphorylation-dependent manner

Since DYRK1A catalyzes the phosphorylation of β-catenin and Hsp90ab1, we investigated whether DYRK1A binds to these two proteins. Coimmunoprecipitation experiments demonstrated that DYRK1A interacts with β-catenin and Hsp90ab1 at endogenous levels in zebrafish embryos (Fig. 3a) and in transfected HEK293T cells (Fig. 3c, e). Furthermore, whole-mount immunostaining of zebrafish embryos revealed colocalization of DYRK1A with both β-catenin and Hsp90ab1, with the majority of the colocalization detected in the cytoplasm (Fig. 3b). Similarly, exogenously expressed DYRK1A and β-catenin/Hsp90ab1 were colocalized in HEK293 cells (Fig. 3d, f). In addition, the interaction between DYRK1A and β-catenin/Hsp90ab1 was validated by an in vitro protein binding assay (Extended Data Fig. 11).

To confirm the requirement of phosphorylation of β-catenin and Hsp90ab1 for the interaction between DYRK1A and β-catenin/Hsp90ab1, we constructed mutants of β-catenin/Hsp90ab1 by replacing serine (S) and threonine (T) with alanine (A). Coimmunoprecipitation and immunostaining in HEK293 cells showed that either mutation abrogated the interaction between these two proteins (Fig. 3c–f and Extended Data Fig. 12). Immunostaining colocalization was analyzed using Olympus confocal microscope software. The parameter “Overlap” is a key index, ranging from −1 to 1, with 1 indicating 100% colocalization. Immunostaining showed that wild-type β-catenin localized to the nucleus and overlapped with DYRK1A (average overlap 0.8419), while the T551A S552A mutant β-catenin localized to the cell membrane and showed decreased overlap with DYRK1A (average overlap 0.1241). Wild-type Hsp90ab1 localized in the cytoplasm and overlapped with DYRK1A (average overlap 0.5378). Although the expressed location of S225A S254A mutant Hsp90ab1 changed to the nucleus, its overlap with DYRK1A was significantly reduced (average overlap 0.1612). In brief, these results suggested the requirement for phosphorylation of β-catenin and Hsp90ab1 for DYRK1A-β-catenin/Hsp90ab1 interaction.

We next determined whether DYRK1A abolished the interaction between Hsp90ab1 and the TGF-β receptor. Coimmunoprecipitation showed that the Hsp90ab1-TGF-β receptor 1 interaction decreased when DYRK1A was overexpressed in HEK293 cells and zebrafish embryos (Fig. 3g and Extended Data Fig. 13). Next, we determined whether the S225 and S254 sites of Hsp90ab1 play an important role in Hsp90ab1-TGF-β receptor interaction. As expected, the S225A and S254A mutant of Hsp90ab1 showed reduced binding to the TGF-β receptor (Fig. 3h). Taken together, DYRK1A inhibited TGF-β signaling by preventing the interaction of Hsp90ab1 with the TGF-β receptor.

### Abnormality of DYRK1A-overexpressed embryos is rescued by tuning up Wnt/TGF-β signaling

Based on the data described above, we hypothesized that DYRK1A-overexpressed embryos could be rescued by regulating Wnt/TGF-β signaling. Since the DYRK1A-overexpressed embryo enhances Wnt/β-catenin and inhibits TGF-β signaling, we synthesized TGF-β ligand *sqt* mRNA and used the Wnt/β-catenin inhibitor ICG-001 to treat DYRK1A-overexpressed embryos. The model embryos injected with 0.1 pg *sqt* mRNA and treated with 10 μm Wnt/β-catenin inhibitor ICG001 simultaneously demonstrated a significant decrease in the occurrence of abnormality; moreover, the body axis of the majority of DYRK1A-overexpressed embryos was restored to normal length (Fig. 4a). Meanwhile, the expression of organizer markers *gsc* and *chd* in the DYRK1A-overexpressed embryo was restored to normal levels (Fig. 4b). Furthermore, we also detected reduced phosphorylation level of DYRK1A and β-catenin and increased phosphorylation level of Smad2 (Extended Data Fig. 14). These results suggest that regulation of Wnt/TGF-β signaling has the potential to treat DS during the embryonic period.

### Molecular abnormalities in the zebrafish DS embryo model are present in amniocytes from fetuses diagnosed with DS and provide an explanation for the abnormality of HSCs in DS

**Fig. 5:**
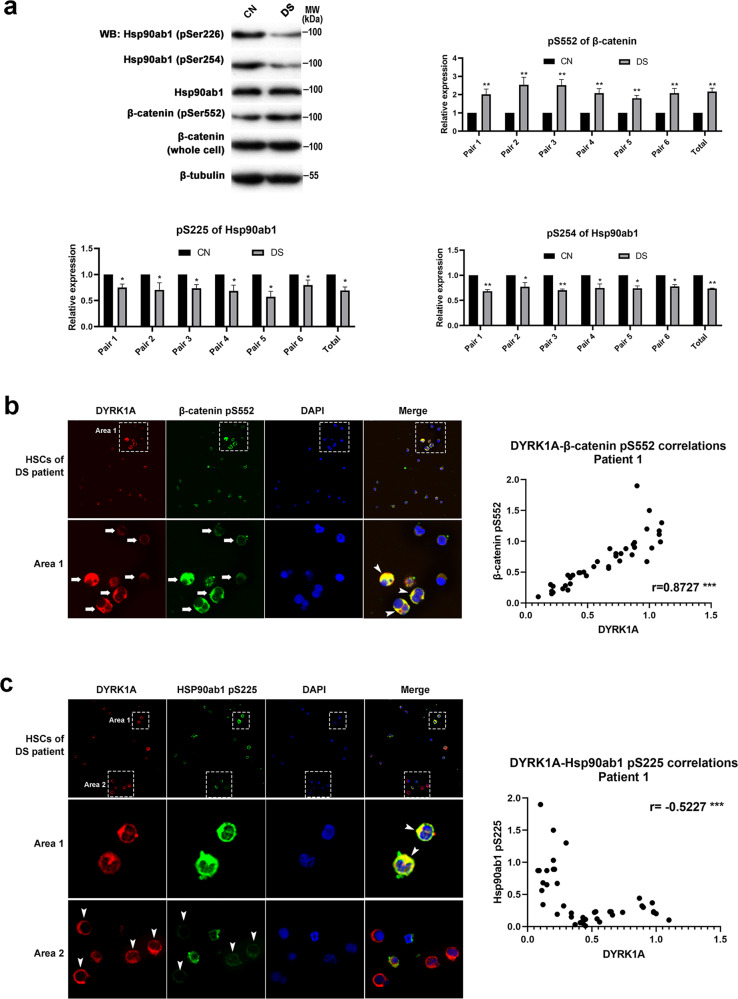
The uncovered molecular mechanism from the zebrafish DS embryo model is conserved in DS patients. **a** Representative western blot assay and corresponding statistics to detect the phosphorylation sites of β-catenin and Hsp90ab1 in amniocytes. **b** DYRK1A expression positively correlated with the phosphorylation level of β-catenin Ser552 site in HSCs from DS patients. Arrows show the detected HSCs, arrowheads show the colocalization of two proteins. **c** DYRK1A expression negatively correlated with the phosphorylation level of the Hsp90ab1 S225 site in HSCs from DS patients. Arrowheads in Area 1 show the colocalization of two proteins, and arrowheads in Area 2 show the detected HSCs.

Due to ethical restrictions, we could not obtain embryos from patients with DS for relevant molecular mechanism verification and therapeutic exploration. Therefore, we collected amniocytes from fetuses diagnosed with DS and performed assays to test the molecular mechanism uncovered by zebrafish DYRK1A-overexpressed embryos in humans. Amniocytes are free-flowing fetal cells present in the amniotic fluid, which originate from all three germ layers of the fetus [27, 28] and are used routinely for prenatal diagnostic purposes, including diagnosis of DS. Consistent with our zebrafish model data, western blotting showed increased phosphorylation of β-catenin and decreased Hsp90ab1 phosphorylation in DS amniocytes (Fig. 5a). We also detected similar expression patterns of Wnt/TGF-β key factors in the DS amniocytes by qPCR. The mRNA levels of *C-MYC* and *LEF1*, well-characterized targets of Wnt/β-catenin signaling [29], were increased in DS amniocytes. Meanwhile, the expression levels of the cyclin-dependent kinase inhibitors p15 and p21, induced by TGF-β and transcription mediated by Smad2/3 [24], decreased (Extended Data Fig. 15).

Consistently, the proteomic work of Cho et al. with amniocytes [28] also showed corresponding regulation of Wnt and TGF-β-related pathways. We found that the expression of Wnt-related proteins, including PLCB4 (ratio 4.6108), SLC9A3R1 (NHERF1, ratio 2.6073), CAMK2G (CAMK-II, ratio 2.2733), PRKACB (PKA C-β, ratio 2.1884), NXN (ratio 1.72), ROCK2 (ratio 1.7158), PPP2CB (PP2Aβ, ratio 1.5833), ROCK1 (ratio 0.56493), DKK3 (ratio 0.29289), and PPP2R1B (ratio 0.26671) (Extended Data Fig. 16a) in their work. The PLCB4 protein (Phospholipase C Beta 4) belongs to a group of enzymes that hydrolyze phospholipids [30]. PLCB4 expression was significantly higher in highly activated Wnt cell lines. Silencing of PLCB4 mRNA using specific siRNAs resulted in a decrease in basal Wnt activity [31]. The sodium-hydrogen antiporter 3 regulator 1 (*SLC9A3R1*) gene consists of six exons and encodes the Na+/H+ exchanger regulatory factor 1 (NHERF1). The PDZ2 domain of NHERF1 selectively stabilized the interaction between β-catenin and E-cadherin. In the absence of NHERF1, the β-catenin/E-cadherin association is disrupted, leading to decreased β-catenin levels at the plasma membrane localization [32]. PRKACB (PKA C-β) is a member of the PKA family of protein kinases (PKA), which phosphorylates residue S675 of β-catenin and enhances its transcriptional activity by promoting its stability [33]. Dickkopf (Dkk)-3, a member of the Dkk family of Wnt antagonists, can reduce the cytoplasmic accumulation of β-catenin in Saos-2 osteosarcoma cells and inhibit TCF-4 activity in PC12 rat pheochromocytoma cells [34]. Rock1 is an effector of RhoA and is involved in multiple cellular functions; β-catenin-overexpressing cardiomyocytes showed decreased ROCK1/PTEN expression both at mRNA and protein levels [35].

The decreased TGF-β-related protein MAPK3 (ratio 0.051538), THBS1 (ratio 0.12663), THBS2 (ratio 0.15078), TGFBI (ratio 0.19756), COMP (ratio 0.2643), PPP2R1B (PP2A-Abeta, ratio 0.26671), TGFB1I1 (ratio 0.39299), CTGF (ratio 0.4179), and ROCK1 (ratio 0.56493) levels can also be found in their results (Extended Data Fig. 16b). MAPK3 is a key factor in Ras-MAPK signaling; TGF-β activates the Ras/MAPK pathway and modulates Smad signaling pathway activation [36]. TGFβ1 induces THBS1 (thrombospondin-1) expression via Smad3, which contributes to invasive behavior during glioblastoma expansion [37]; TGF-β also stimulates both THBS1 and CISP/THBS2 synthesis by bovine adrenocortical cells [38]. In contrast, TGF-β activation by THBS1 is both required and sufficient for the development of PH in Schistosoma-exposed mice [39] and in recessive dystrophic epidermolysis bullosa fibroblasts [40]. TGFBI (transforming growth factor beta-induced) is the gene with the most reduced expression in the taxane-resistant line; it is an extracellular matrix protein whose secretion is induced by TGF-β1 stimulation. Its functions include cell adhesion to the ECM and integrin-mediated signaling [41]. The cartilage oligomeric matrix protein (COMP) is an important non-collagenous cartilage protein that is essential for the structural integrity of the cartilage extracellular matrix. COMP-bound TGF-β increases TGF-β-dependent transcription and enhances bioactivity [42]. Protein phosphatase 2A (PP2A) has been shown to specifically interacts with activated TβRI, and PP2A is a component of a TGF-β-induced signal transduction pathway that controls protein translation and G1/S progression [43]. TGFB1I1 (transforming growth factor-β1-induced transcript 1) is a TGF-β-responsive gene involved in the cellular response to vascular injury [44]. CTGF (connective tissue growth factor) is constitutively expressed in activated hepatic stellate cells and acts downstream of TGF-β to modulate extracellular matrix production in the fibrotic liver [45]. TGF-β1 has been shown to upregulate CTGF expression in rat and hen granulosa cells [46]. ROCK1 belongs to the Rho/Rho‑associated coiled‑coil‑forming protein kinase (Rock) pathway; the TGF-β/Smad inhibitor downregulates RhoA, RhoC, and Rock1 expression in the process of transforming lung fibroblasts to myofibroblasts [47]. Taken together, these results indicate that our established DS zebrafish embryo model is suitable for DS research, with molecular mechanisms conserved between zebrafish and humans.

It has been reported that DS fetal liver exhibits expansion of HSC numbers, and in vitro purified trisomy 21 fetal liver HSCs have erythroid megakaryocyte-biased gene expression together with reduced expression of lymphoid genes. Consistent with this, fetal liver HSC function is also markedly abnormal in DS. In particular, fetal liver HSCs generate more megakaryocytes and erythroid cells [18, 48]. Importantly, Wnt and TGF-β signaling is essential for HSC homeostasis [49].

Similar to the characteristics of HSCs in DS, our zebrafish model exhibited increased expression of the hematopoietic progenitor marker *scl*; the erythroid progenitor marker *gata1* at the 8-somite stage during primitive hematopoiesis; significantly increased the expression level of the hematopoietic stem-cell markers *runx1* at 28 hpf and *cmyb* at 48 hpf during definitive hematopoiesis and reduced expression levels of the T lymphocyte markers *rag1* and *rag2* at 4 dpf (Extended Data Fig. 4). Therefore, the similarity of our zebrafish model to DS prompted us to ask whether the mechanism observed in zebrafish also functions in the hematopoietic system of DS patients.

Next, we collected peripheral blood from patients diagnosed with DS and isolated HSCs. Based on the care of patients, we used the remaining peripheral blood after diagnosis for related experimental detection, so the number of isolated HSCs was not sufficient for flow cytometry and western blotting. Therefore, we used immunofluorescence (IF) on cell smears for detection and then finally counted the cell content.

As expected, we detected a positive correlation between DYRK1A expression and the phosphorylation level of the β-catenin Ser552 residue (Fig. 5b and Extended Data Fig. 17a). Meanwhile, DYRK1A expression negatively correlated with the phosphorylation level of Hsp90ab1 residue S225 in isolated HSCs from children with DS (Fig. 5c and Extended Data Fig. 17b). These results were similar to those obtained from the zebrafish DS embryo model, suggesting a conserved molecular mechanism of impairment of TGF-β/Wnt signaling.

Next, we treated isolated DS HSCs with 2 ng/ml TGF-β ligand recombinant TGF-β1 protein and 10 μm Wnt/β-catenin inhibitor ICG-001 simultaneously. Encouragingly, we detected a reduced proliferation of HSCs using the pH3 antibody (Extended Data Fig. 18). Taken together, these results suggest that regulation of TGF-β/Wnt signaling changes the abnormal status of DS HSCs, verifying the molecular mechanism characterized in the zebrafish model.

## Discussion

Despite the high prevalence of DS and early identification of its cause, its molecular pathogenesis is poorly understood, with specific treatments practically unavailable [28]. The current approach to understanding abnormal multi-system and multi-organ systems, such as those observed with DS, is to study abnormalities of a certain system or organ, such as the nervous, reproductive, or circulatory systems. Such research is unlikely to reveal the root causes and molecular mechanisms of the abnormal development of multiple systems and multiple organs. Our research attempts to study the causes and molecular mechanisms of DS multi-system and multi-organ abnormalities from the perspective of early embryonic organizer formation.

According to the gene-dosage hypothesis and haploinsufficiency rank, although HSA21 contains more than 300 genes, only a subset contributes to DS phenotypes. Interestingly, the DS critical region genes implicated in gene-dosage mechanisms are wild-type genes; thus, it is only an increase of 50% in expression that produces overall effects [13]. Several studies have shown that in some tissues in mouse models, increased expression of a single additional allele within Hsa21, *Dyrk1a*, for example, is sufficient to produce changes in stem/progenitor cell behavior, which correlates with clinical phenotypic observations, such as leukemia and cognitive defects [18].

Overexpression of *Dyrk1a* in mice leads to important behavioral impairments, cognitive deficits, motor abnormalities, neuronal alterations, retinal defects, vascular defects, gastrointestinal tract abnormalities, immune response defects, abnormal maturation of the thymus, and impaired function of T lymphocytes [13–15] that recapitulate those associated with human trisomy 21, suggesting that overexpression of this kinase is sufficient to produce this phenotype in an otherwise normal, diploid genetic background. Therefore it can be considered the cause of the observed DS phenotype [16, 17].

It is particularly noteworthy that we found that the key protein β-catenin of the Wnt pathway has abnormally increased phosphorylation at residues T551 and S552. Increased phosphorylation of S552 has been shown to enhance β-catenin transport into the nucleus and downstream transcription levels [50], while the function of phosphorylation of T551 has not yet been reported. In addition, the phosphorylation of residues S225 and S254 of the key regulatory protein Hsp90ab1 in the TGF-β pathway was abnormally reduced. To date, only the phosphorylation of these two sites and the AhR complex [51] and P-gp expression [52] are related, and there is no report of interaction with the TGF-β receptor. In this study, we demonstrated that phosphorylation at four sites in β-catenin and Hsp90ab1 were modified by DYRK1A and likely play a critical role in protein interaction with DYRK1A, providing a new molecular regulation for signal transduction.

Taken together, we attempted to understand the molecular pathogenesis of DS from the perspective of embryonic organizer formation and body axis development, to provide an explanation as to why a number of diverse medical conditions are associated with DS. Based on the gene-dosage theory and the *Dyrk1a*^*+/++*^ mouse model, we established a DYRK1A-overexpressed model in zebrafish. Our data are summarized graphically as altered Wnt and TGF-β signaling through changes in β-catenin and Hsp90ab1 phosphorylation (Extended Data Fig. 19). Notably, combined Wnt and Nodal signaling can induce a human organizer [53]. Verification of the molecular mechanism in human DS amniocytes and isolated HSCs suggests mechanistic conservation between zebrafish and humans and the utility of our zebrafish model. Most importantly, the simultaneous alteration of Wnt and TGF-β signaling may present a new therapeutic target for DS.

## Supplementary information

Methods+Extended data Fig 1-19

Supplementary Table 1-4

original data files

Reproducibility checklist

## Data Availability

The data underlying this article will be shared on reasonable request to the corresponding author.
