# Supplementary material for: Embryonic organizer formation disorder leads to multiorgan dysplasia in Down syndrome
Source: Cell Death Dis. 2022 Dec 19;13(12):1054. doi: 10.1038/s41419-022-05517-x (PMC9763398; doi:10.1038/s41419-022-05517-x)
Supplement: Supplementary file 1 — Methods+Extended data Fig 1-19 [file 41419_2022_5517_MOESM1_ESM.pdf]

Methods

Ethical approval and Ethics Statement.

All experiments in this study were in accordance with the "Guide for the Care and Use of Laboratory Animals" (Eighth Edition, 2011. ILARCLS, National Research Council, Washington, D.C.) and were approved by the Animal Care and Use Committee of West China Second University Hospital, Sichuan University (Approval ID: HXDEYY20131021).

Amniocytes and peripheral blood samples of Down syndrome (DS) patients and control were obtained from West China Second University Hospital. The work on patients' samples was approved by the above medical institutions (Approval ID: 2019, No. 34). Written informed consent was obtained from the patients and healthy control donors before drawing blood samples. All procedures performed in studies involving human participants were in accordance with the ethical standards of the institutional and/or national research committee and with the 1964 Helsinki declaration and its later amendments or comparable ethical standards.

Zebrafish lines and Embryos.

Wildtype (WT) AB strain, *Tg(flk1:GFP)* and *Tg(gsc:GFP)* fish lines were utilized. Staging of the embryos was carried out as previously described [1].

Reagents and antibodies.

ICG-001 (HY-14428) was from MCE. Animal-Free Recombinant Human TGF-β1 (AF-100-21C) was from PeproTech. Normal rabbit IgG (sc-2027) and normal mouse IgG (sc-2025) were from Santa Cruz. The following antibodies were used: anti-c-Myc (Santa Cruz, sc-40), anti-HA (Santa Cruz, sc-805), anti-HA (ABclonal, AE008), anti-

His (Proteintech, 66005-1-Ig), anti-β-catenin (Cell Signaling Technology, 9562), anti-β-catenin (Proteintech, 66379-l-lg),anti-β-catenin (pSer552) (Biorbyt, orb336008), anti-β-tubulin (Zen Bioscience, 200608), anti-DYRK1A (Absin, abs121442a), anti-DYRK1A (Santa Cruz, sc12568), anti-DYRK1A (phospho-Tyr321/273, Biorbyt, orb714399), anti-Hsp90ab1 (Zen Bioscience, 220007), anti-Hsp90ab1 (Proteintech, 11405-1-AP), anti-Hsp90ab1 (pSer226) (Biorbyt, orb393321), anti-Hsp90ab1 (pSer254) (Biorbyt, orb159735), anti-Histone H2B (Huabio, ET1612-25), anti-Smad2 (Cell Signaling Technology, 5339), anti-p-Smad2 (Cell Signaling Technology, 3108), anti-Smad3 (Cell Signaling Technology, 9523), anti-p-Smad3 (Cell Signaling Technology, 9520), anti-TβR I (TGFBR1, ABclonal, A16983), anti-TβR II(TGFBR2, ABclonal, A1415), anti-pH3 (Phospho-Histone H3 Ser10, Beyotime, AF1180), anti-GFP (CMCTAG, AT0028).

Constructs and microinjection.

Human *DYRK1A* full length coding sequence (cds) was amplified from K562 cell line, zebrafish *dyrk1a* coding region sequence was from Dharmacon (Catalog Number MDR1734-202804618). Expression plasmids of Hsp90ab1, TBRI, TBRII and β-catenin were obtained from Addgene (#22487, #19161, #11766 and #17199). Myc/HA-tag coding sequence was added to upstream of cds respectively, and fused sequences were cloned into pcDNA3.1+ vector (Invitrogen) for capped mRNAs synthesis and transfection. Primary plasmids of Gal4-UAS system are CZP06 and CZP08 from China Zebrafish Resource Center (CZRC). There were 10ng/μl of DNA constructs and

10ng/μl of Tol2 mRNA that were injected into one-cell stage embryos.

Reagent treatment of zebrafish embryos

Embryos (20 embryos in a well of 6-well plate with 3 ml culture water) were treated with DMSO or Wnt inhibitor ICG-001 (10μM) for 2 hours at indicated stage and then subject to following analysis.

Amniotic fluid cell culture

A total of six DS (Down syndrome) and six CN (Chromosomally normal) amniocyte samples were collected by amniocentesis from women at 18 to 28 weeks of gestation, undergoing prenatal diagnosis. These amniotic fluid cells were a fraction of the cells obtained for cytogenetic analysis and chromosome micro-array (CMA). Before cytogenetic analysis and CMA, all samples were subjected to quantitative fluorescent polymerase chain reaction (QF-PCR) to rapidly detect abnormal numbers of chromosomes 13/18/21 and sex chromosome. 21-Trisomy cells (DS) and CN cells were grown in T-12.5 cm^2 flasks for approximate 10 to 14 days in medium BIO-AMF ™-3 (complete culture medium for human amniotic fluid cells and chorionic villi samples) (Biological Industries, Ref 01-196-1B). Then we harvested the cells for western blot and qPCR assay. Cells from an individual constituted a single sample without pooling at any step. The study protocol was approved by West China Second University Hospital. Informed consent was obtained from all participants. The study was performed in accordance with the Declaration of Helsinki Principles.

DS children peripheral blood collection and HSCs separation

Human peripheral blood cells were separated by kit with procedure provided by manufacture (LGS106805, TBDsciences, Tianjin, China). Then，human HSCs were separated using EasySep™ Human Cord Blood CD34 Positive Selection Kit II (STEMCELL Technologies Inc.).

Assays and statistics

Quantitative phosphoproteome analysis of embryos was performed by PTM-Biolabs (HangZhou) Co., Ltd. Zebrafish embryo whole-mount in situ hybridization, immunofluorescence in zebrafish embryos, immunofluorescence in cells, in vitro synthesis of RNA, microinjection, subcellular fractionation, western blotting, immunoprecipitation, cell culture, transfection, luciferase reporter assays, quantitative real time RT–PCR (qRT–PCR) analysis **(Supplementary Table 4 see primers)**, in vitro protein synthesis/binding assay, grayscale measurement, immunostaining colocalization analysis, point mutation expression plasmids construction and statistics were performed as previously described[1]. qPCR and luciferase reporter assays with three independent biological replicates and three technical replicates were performed and counted. Western blotting was performed with three independent biological replicates, X-ray films or Digital images (by ChemiDoc MP Imaging system, Bio-Rad) was used to get bands, and the gotten bands were measured by grayscale. The immunofluorescence in zebrafish embryos and cells was photographed with Olympus

FV1000 or Olympus FV3000 laser scanning confocal microscope.

Reference

1 Sun, H. *et al.* CFTR mutation enhances Dishevelled degradation and results in impairment of Wnt-dependent hematopoiesis. *Cell Death Dis* **9**, 275, doi:10.1038/s41419-018-0311-9 (2018).

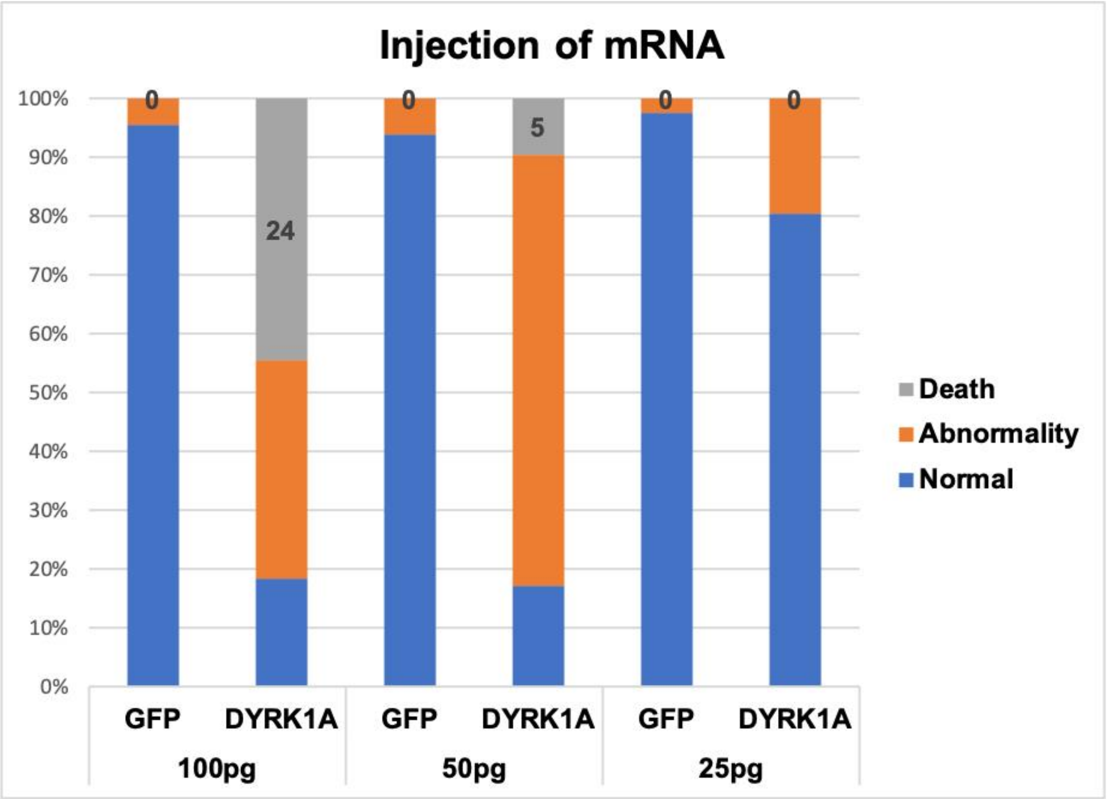

Extended Data Fig. 1 Gene-dosage effect of *DYRK1A* gene on zebrafish embryo development. High doses of *DYRK1A* mRNAs (100pg) produced high rate of embryonic death and severe embryonic deformities. The toxicity of overexpressed *DYRK1A* gene decreased with reduced does of mRNA injection. Number on the histogram indicates the death.

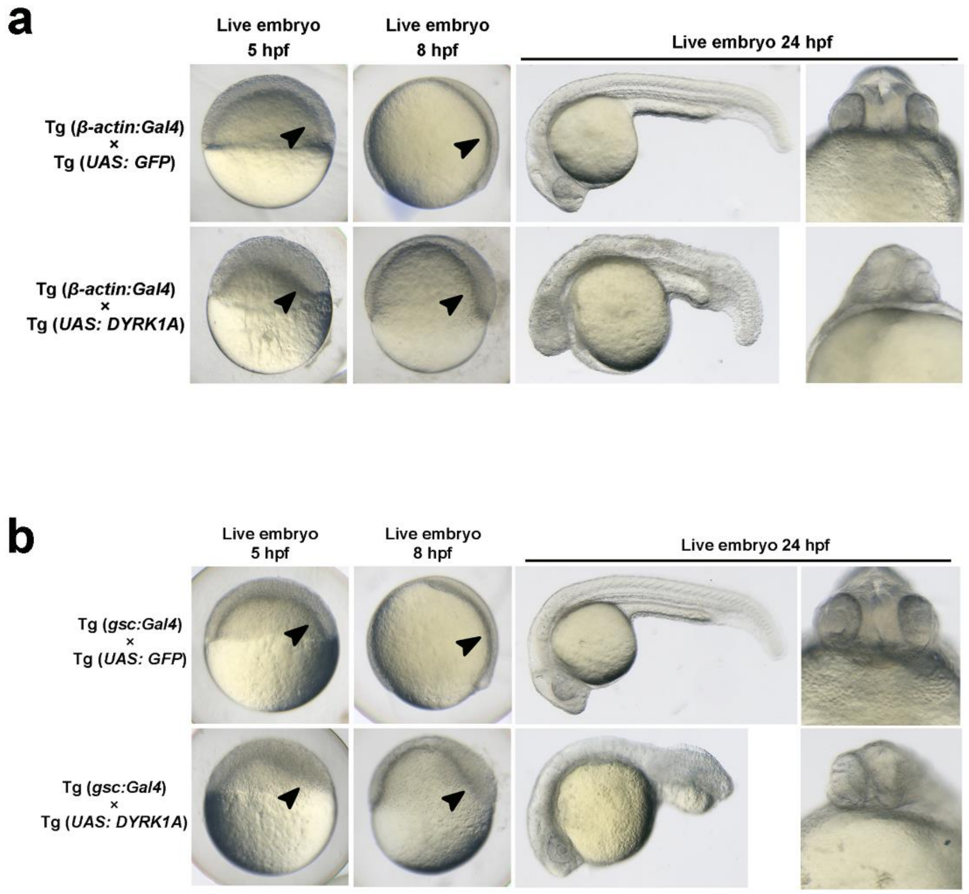

Extended Data Fig. 2 *DYRK1A* overexpression by gal4-UAS system also led to severe embryonic deformities consistent with the result of microinjection of *DYRK1A* mRNAs. UAS: DYRK1A driven by the β-actin **(a)** or gsc **(b)** promoter in zebrafish embryos.

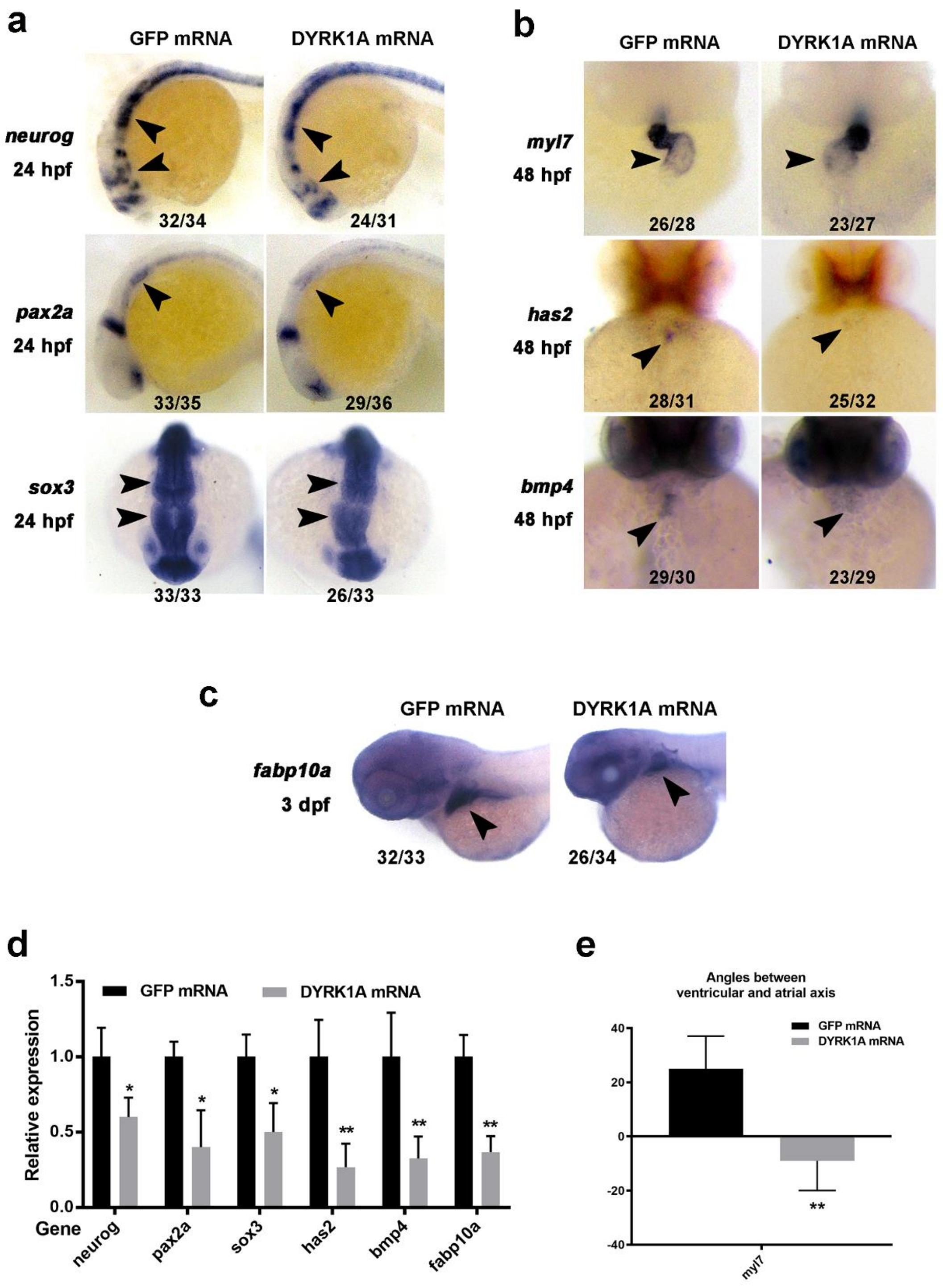

Extended Data Fig. 3 Our established DYRK1A-overexpressed embryonic model demonstrated significant dysplasia of nerve, heart and gut system.

(a) Nervous system marker genes expression at 24 hpf. Embryo orientation: neurog and pax2a, lateral views with the anterior at the left; sox3, head views with the anterior at the bottom. **(b)** Heart morphology (marked by the pan-cardiomyocyte marker myl7) at 48 hpf was impaired significantly in DYRK1A-overexpressed embryonic model. The

expression of bmp4 and has2 is dramatically reduced in DYRK1A-overexpressed embryonic model embryos at 48 hpf. Embryo orientation: ventral views with the anterior at the top. Arrows in myl7 pictures point to the atrium; Arrows in has2 and bmp4 pictures point to the signal generated by detected marker genes. **(c)** WISH results of fabp10a at 72 hpf. Embryo orientation: lateral views with the anterior at the left. **(d)** Histogram representing the relative expression of the detected marker gene. **(e)** Looping angle is represented by the histogram. Measurement for cardiac looping angle at 48hpf was done as our previous description (https://doi.org/10.1016/j.mod.2020.103627).

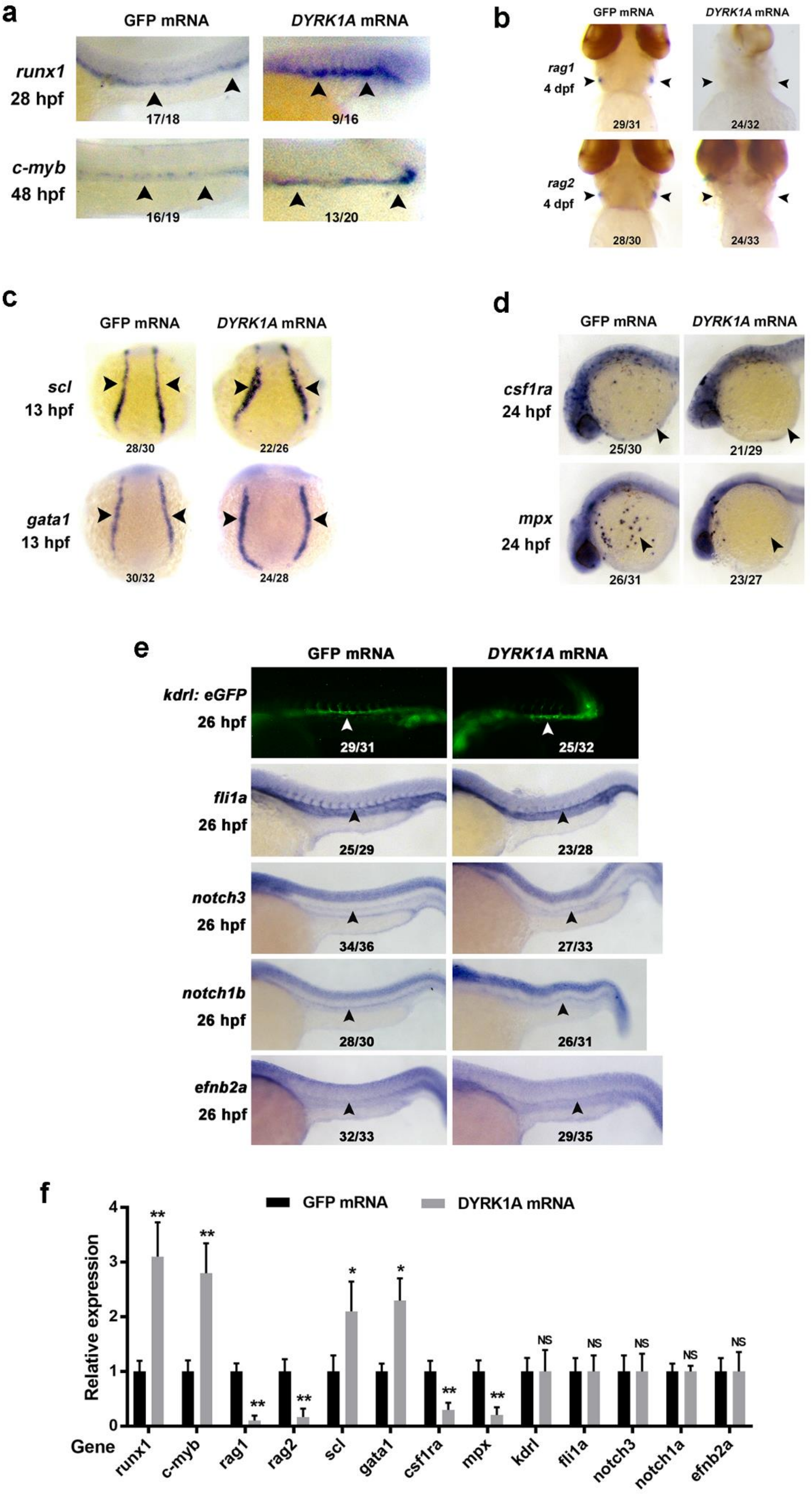

a
GFP mRNA
DYRK1A mRNA
runx1
28 hpf
17/18
9/16
c-myb
48 hpf
16/19
13/20
b
GFP mRNA
DYRK1A mRNA
rag1
4 dpf
29/31
24/32
rag2
4 dpf
28/30
24/33
c
GFP mRNA
DYRK1A mRNA
scl
13 hpf
28/30
22/26
gata1
13 hpf
30/32
24/28
d
GFP mRNA
DYRK1A mRNA
csf1ra
24 hpf
25/30
21/29
mpx
24 hpf
26/31
23/27
e
GFP mRNA
DYRK1A mRNA
kdrl: eGFP
26 hpf
29/31
25/32
fli1a
26 hpf
25/29
23/28
notch3
26 hpf
34/36
27/33
notch1b
26 hpf
28/30
26/31
efnb2a
26 hpf
32/33
29/35
f
GFP mRNA
DYRK1A mRNA
Relative expression
0
1
2
3
4
**
**
**
**
*
*
**
**
NS
NS
NS
NS
NS
Gene
runx1
c-myb
rag1
rag2
scl
gata1
csf1ra
mpx
kdrl
fli1a
notch3
notch1a
efnb2a

Extended Data Fig. 4 DYRK1A-overexpressed embryonic model also demonstrated significant dysplasia of hematopoietic system.

(a) DYRK1A-overexpressed embryo showed enhanced expression of hematopoietic stem cells marker gene *runx1* and *c-myb*. **(b)** Reduced expression of T cell marker genes rag1 and rag2 in DYRK1A-overexpressed embryo. **(c)** DYRK1A-overexpressed embryo also showed enhanced expression of hematopoietic progenitor cells marker gene *scl* and *gata1*. **(d)** Macrophages marker gene *csf1ra* and primitive neutrophils marker gene *mpx* showed reduced expression pattern. **(e)** Expression of the arterial markers kdrl, fli1a, notch3, notch1b and efnb2a did not show obvious change in DYRK1A-overexpressed embryo. Arrowheads denote the caudal artery (CA). **(f)** Histogram representing the relative expression of the detected marker gene.

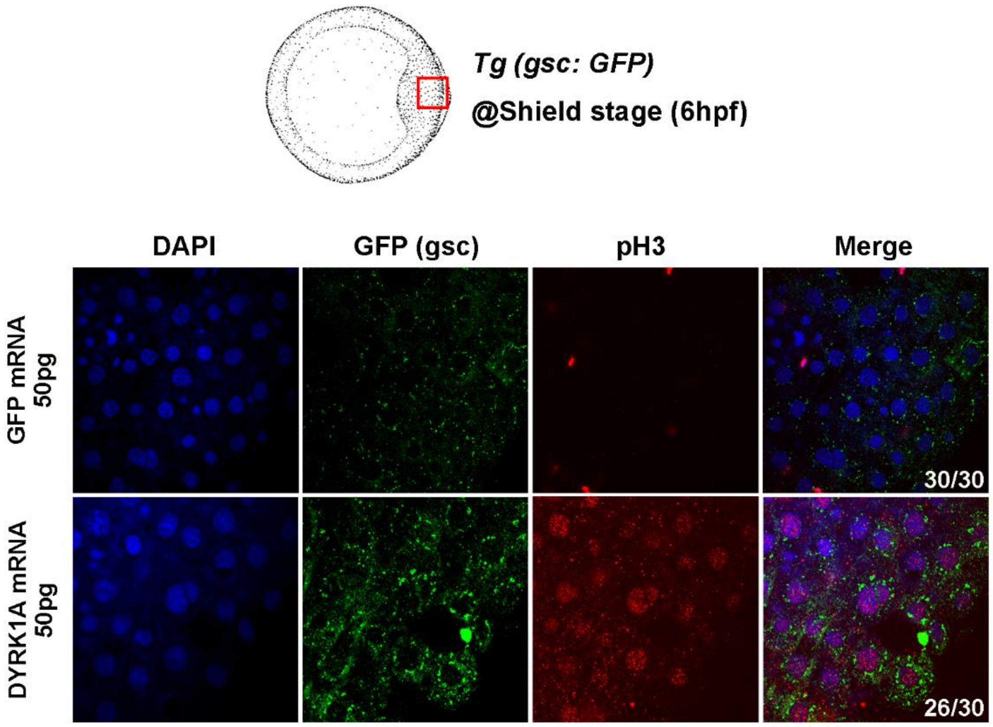

Extended Data Fig. 5 DYRK1A-overexpressed embryo model enhances the proliferation of organizer cells. Embryos were flat-mounted and double labeled with GFP and phosphor-histone H3 antibody.

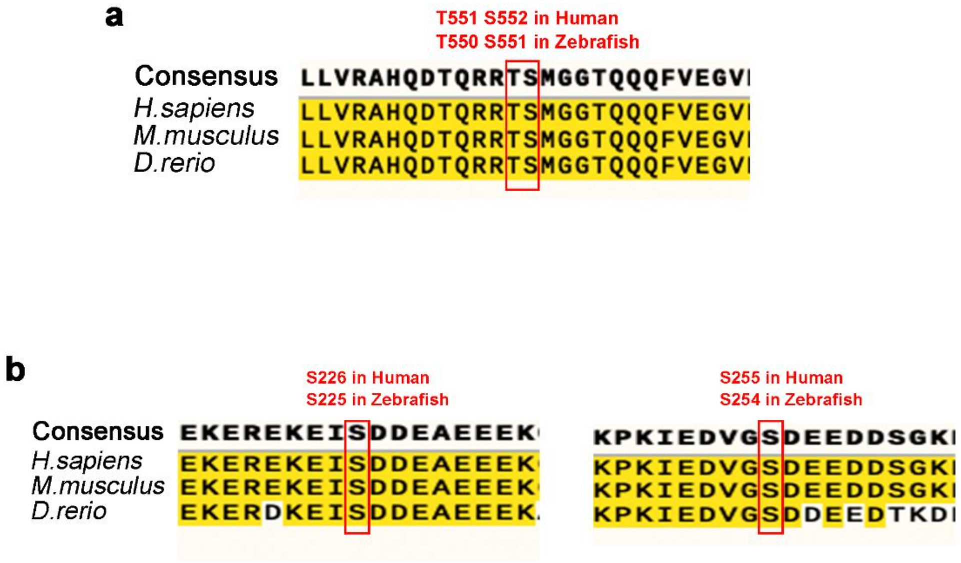

Extended Data Fig. 6 Localization and conservation of amino acid residues of T550 and S551 in β-catenin protein (a), S225 and S254 in Hsp90ab1 protein (b).

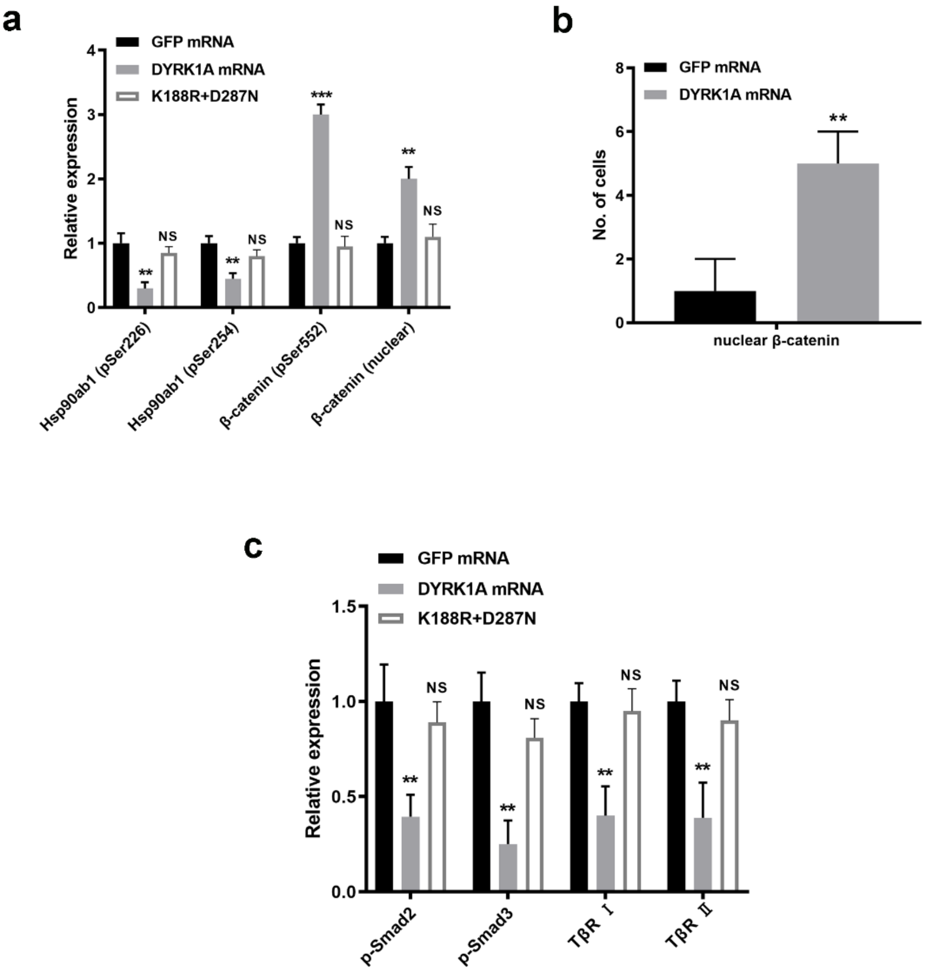

Extended Data Fig. 7 Statistics of Fig 2c (a), Fig 2d (b) and Fig 2e (c).

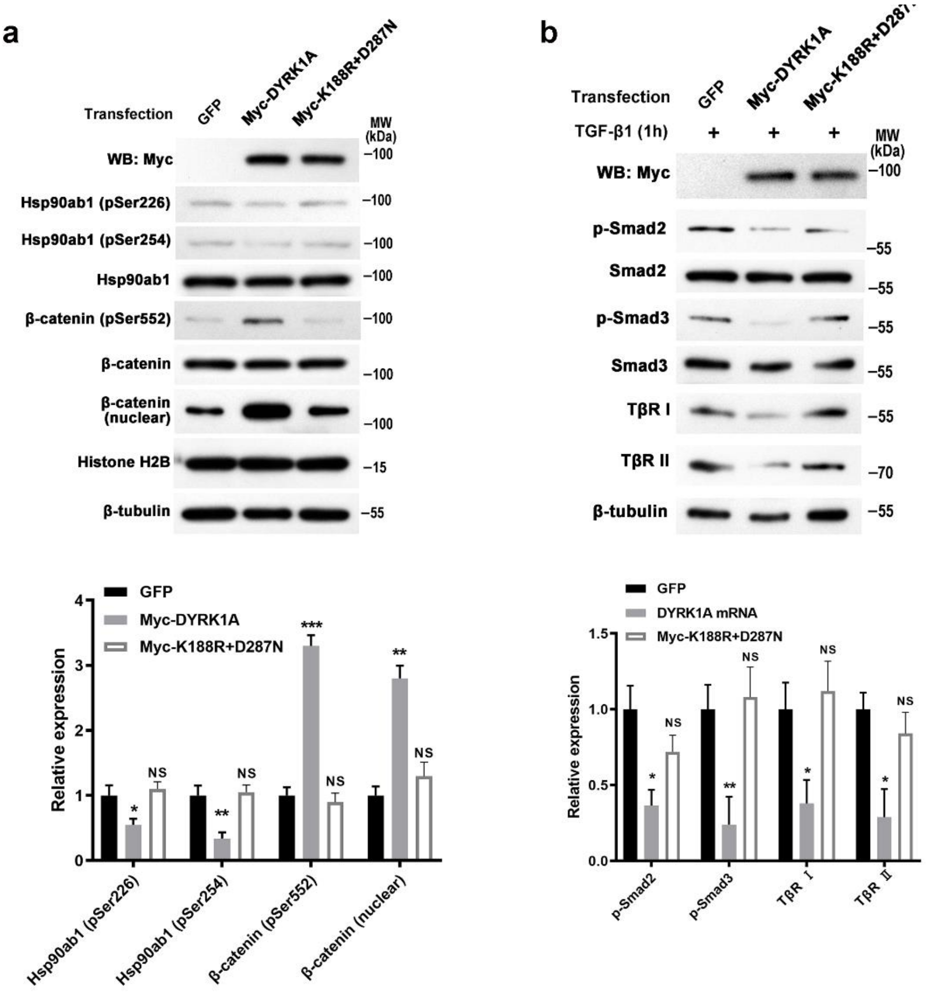

Extended Data Fig. 8 DYRK1A overexpression in vitro in HEK293 cells also regulates the phosphorylation sites of β-catenin and Hsp90ab1 and related Wnt/TGF-β signaling.

(a) Regulation of phosphorylation sites of β-catenin and Hsp90ab1 was verified in vitro using western blot assay in HEK293 cells. **(b)** Overexpressed DYRK1A in HEK293 cells also inhibits Smad2/3 phosphorylation and TGF-β receptor detected by western blot.

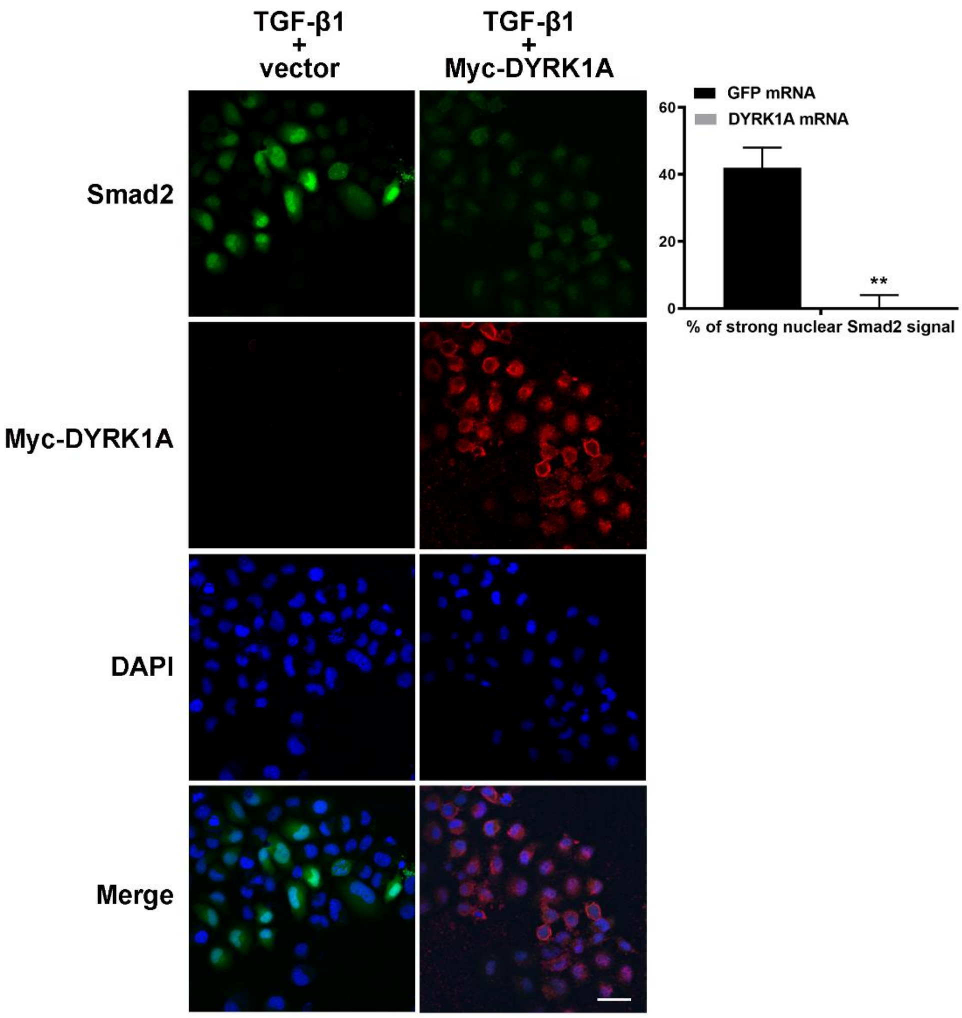

Extended Data Fig. 9 Nuclear enrichment of Smad2 impaired under DYRK1A overexpression. Confocal immunofluorescence (IF) microscopy images of HEK293 cells. Cells were stimulated with TGF-β1 (1 hour) as indicated. IF stainings with Smad2 and Myc antibody. Scale bar represents 20 mm.

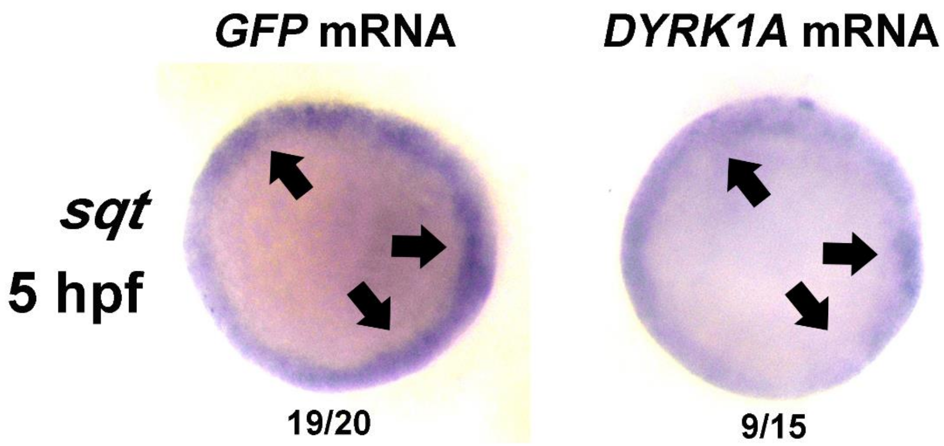

Extended Data Fig. 10 DYRK1A-overexpressed embryo model reduces the expression of TGF-β legend *sqt* by WISH assay. Embryo orientation: animal pole views with the dorsal to the right.

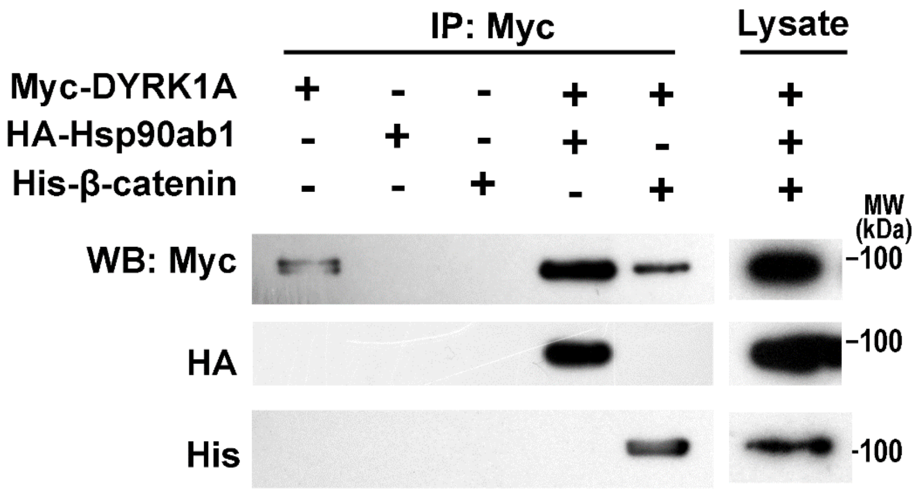

Extended Data Fig. 11 In vitro binding assay identifies the physical interaction of DYRK1A with β-catenin/Hsp90ab1.

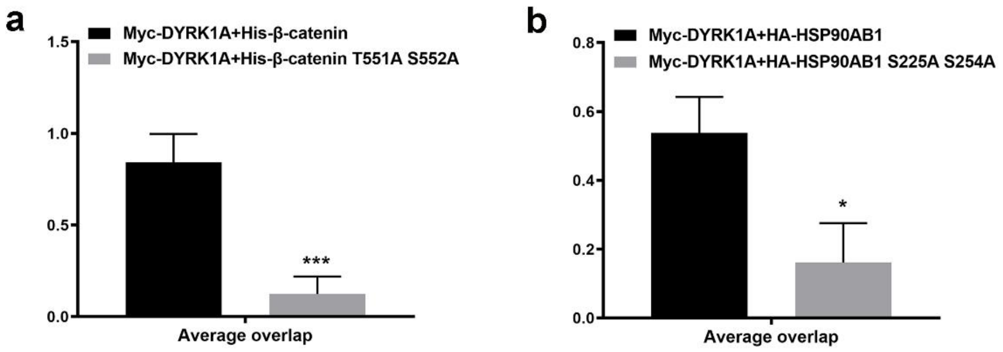

Extended Data Fig. 12 Statistics of Fig 3d (a) and Fig 3f (b).

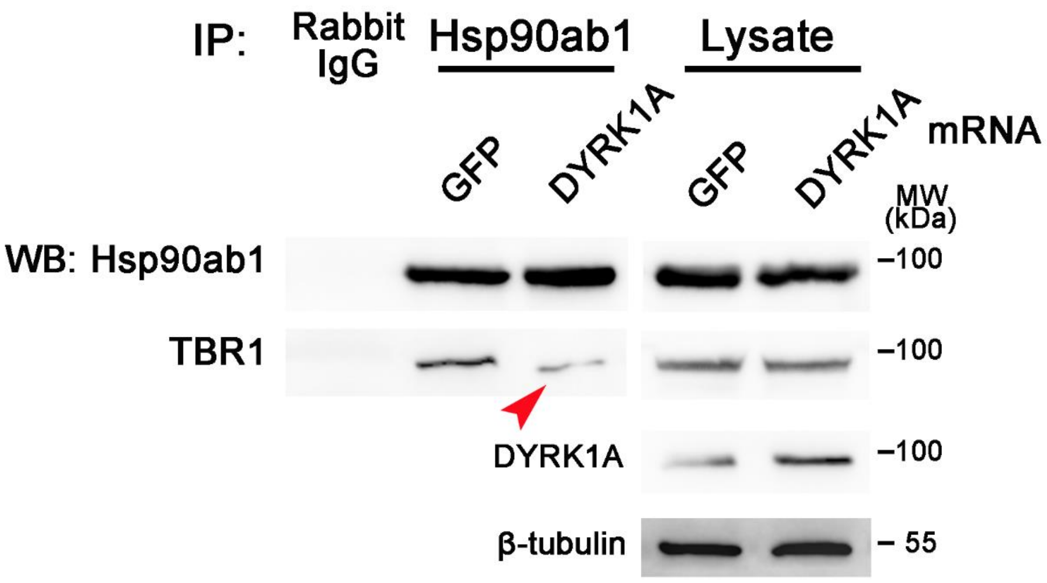

Extended Data Fig. 13 Co-immunoprecipitation (Co-IP) of endogenous DYRK1A and β-catenin/Hsp90ab1 in zebrafish embryos. Co-IP/western blot analysis in zebrafish embryos shows that DYRK1A overexpression decreases the protein–protein interaction between Hsp90ab1 and TGF-β receptor.

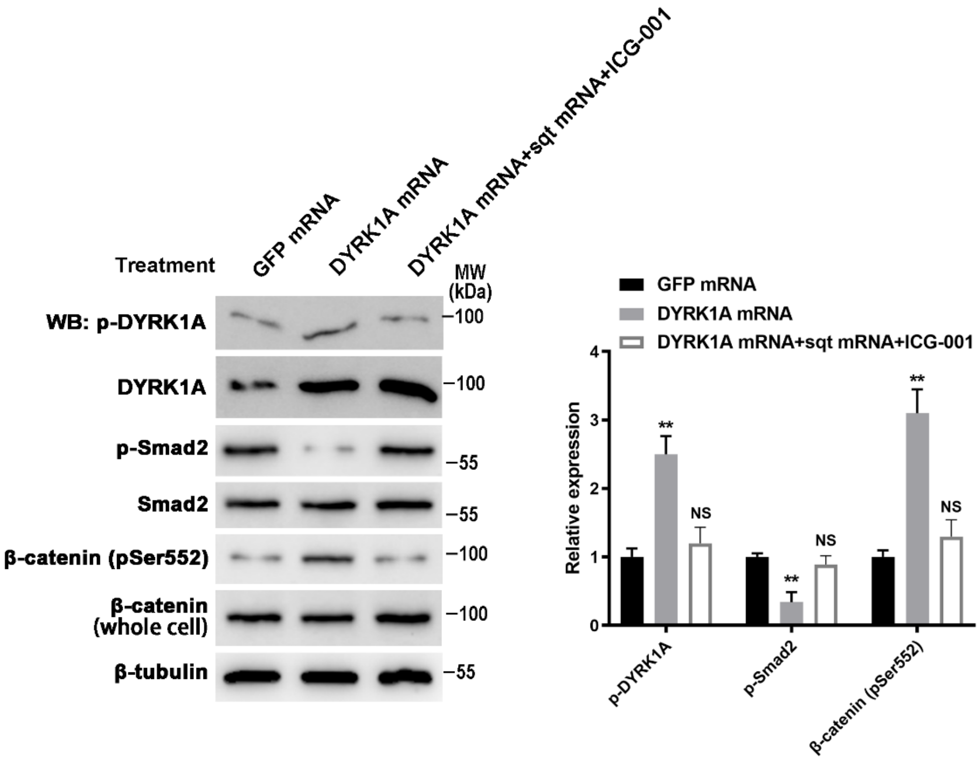

Extended Data Fig. 14 Effects of sqt+ICG001 on phospho-DYRK1A, Wnt/β-catenin and TGF-β activation when treating the DYRK1A-overexpressed embryos.

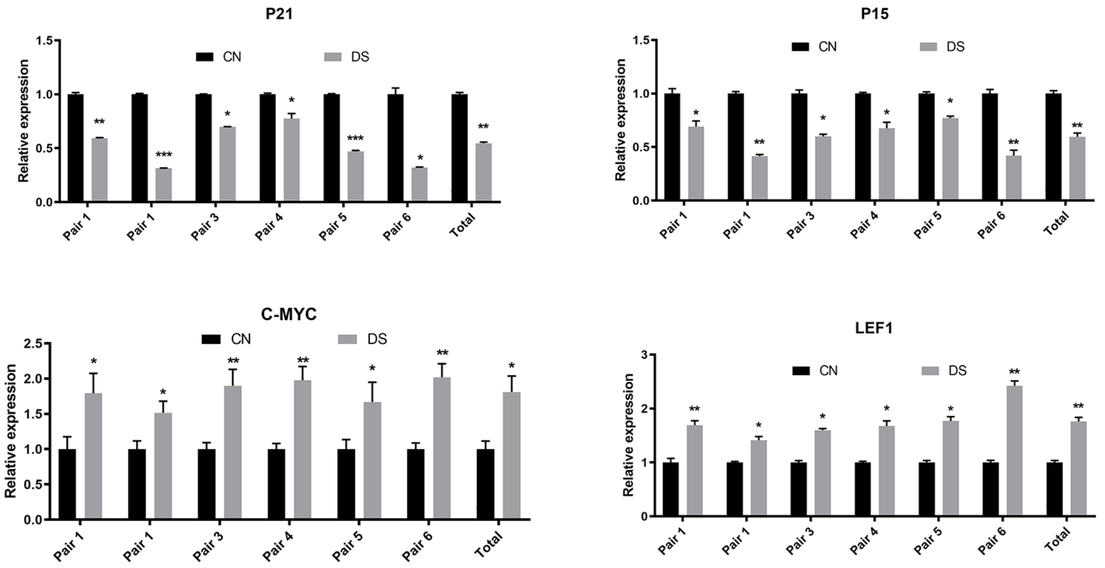

Extended Data Fig. 15 Quantitative real time RT–PCR (qPCR) assay results showed increased expression of Wnt target gene c-myc and lef1 and decreased expression of p21 and p15 mediated by TGF-β in amniocytes.

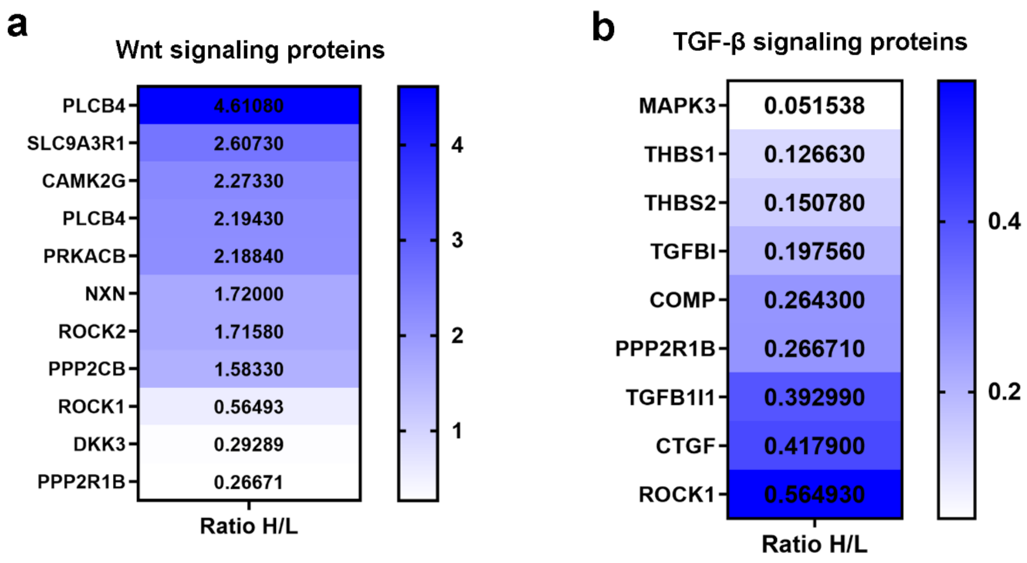

Extended Data Fig. 16 Quantitative proteomic analysis of amniocytes in Cho et al's work also showed the corresponding regulation of key factor of Wnt (a) and TGF-β (b) signaling.

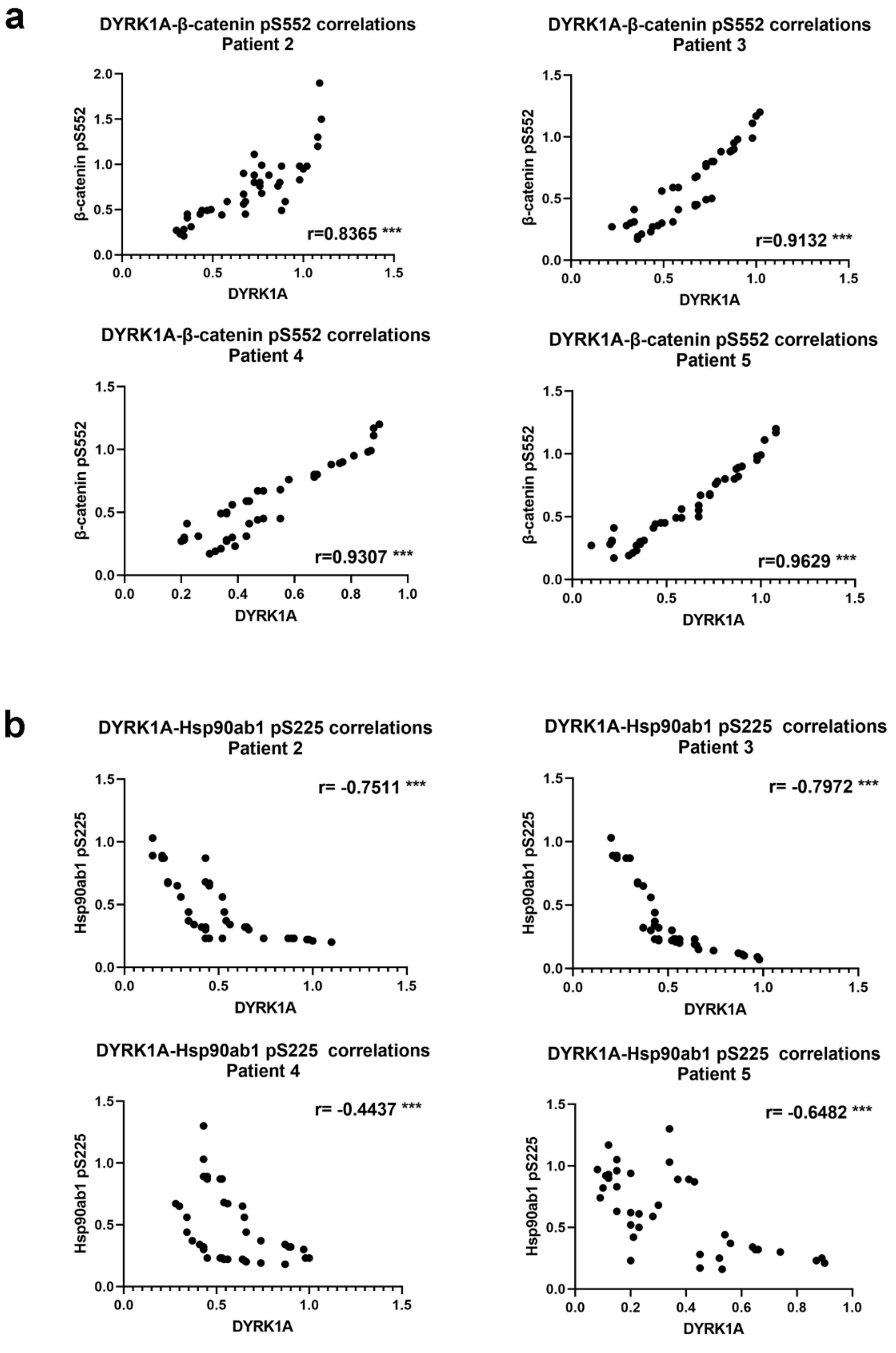

Extended Data Fig. 17 Correlation between DYRK1A and β-catenin/Hsp90ab1 phosphorylation sites in DS isolated HSCs. (a) DYRK1A expression positively correlated with the phosphorylation level of β-catenin Ser552 site in HSCs from DS patients. **(b)** DYRK1A expression negatively correlated with the phosphorylation level of Hsp90ab1 S225 site in HSCs from DS patients.

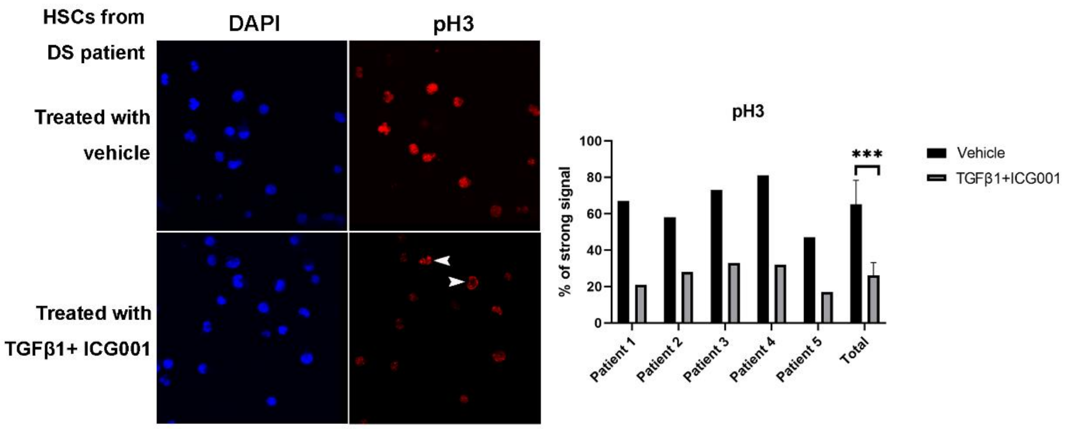

Extended Data Fig. 18 Proliferation of DS HSCs was inhibited by treatment with TGF-β ligand recombinant TGF-β1 protein and Wnt/β-catenin inhibitor ICG-001 simultaneously. The isolated DS HSCs were cultured in RPMI 1640 medium supplemented with 10% fetal bovine serum (FBS) at 37 °C in a 5% CO2 atmosphere and treated with TGF-β1 and ICG-001 immediately for 1 hour. The treated HSCs were collected by centrifugation and smeared on glass slide for immunofluorescence. This assay was performed by staining with pH3 antibody. Arrowheads show the reduced expression of pH3 signal.

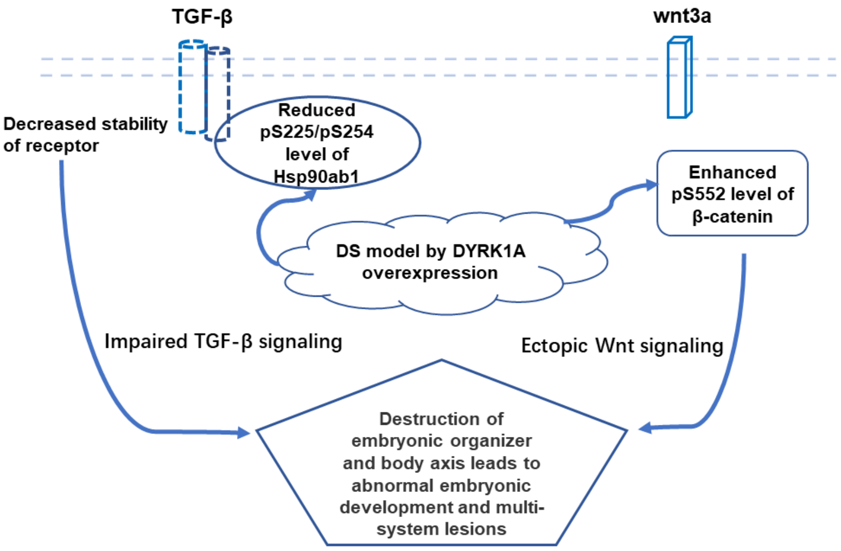

Extended Data Fig. 19 Model for regulation of Wnt and TGF-β signaling by DS in organizer formation and body axis development.
