# Supplementary material for: Embryonic organizer formation disorder leads to multiorgan dysplasia in Down syndrome
Source: Cell Death Dis. 2022 Dec 19;13(12):1054. doi: 10.1038/s41419-022-05517-x (PMC9763398; doi:10.1038/s41419-022-05517-x)

Fig 2C WB Full scan

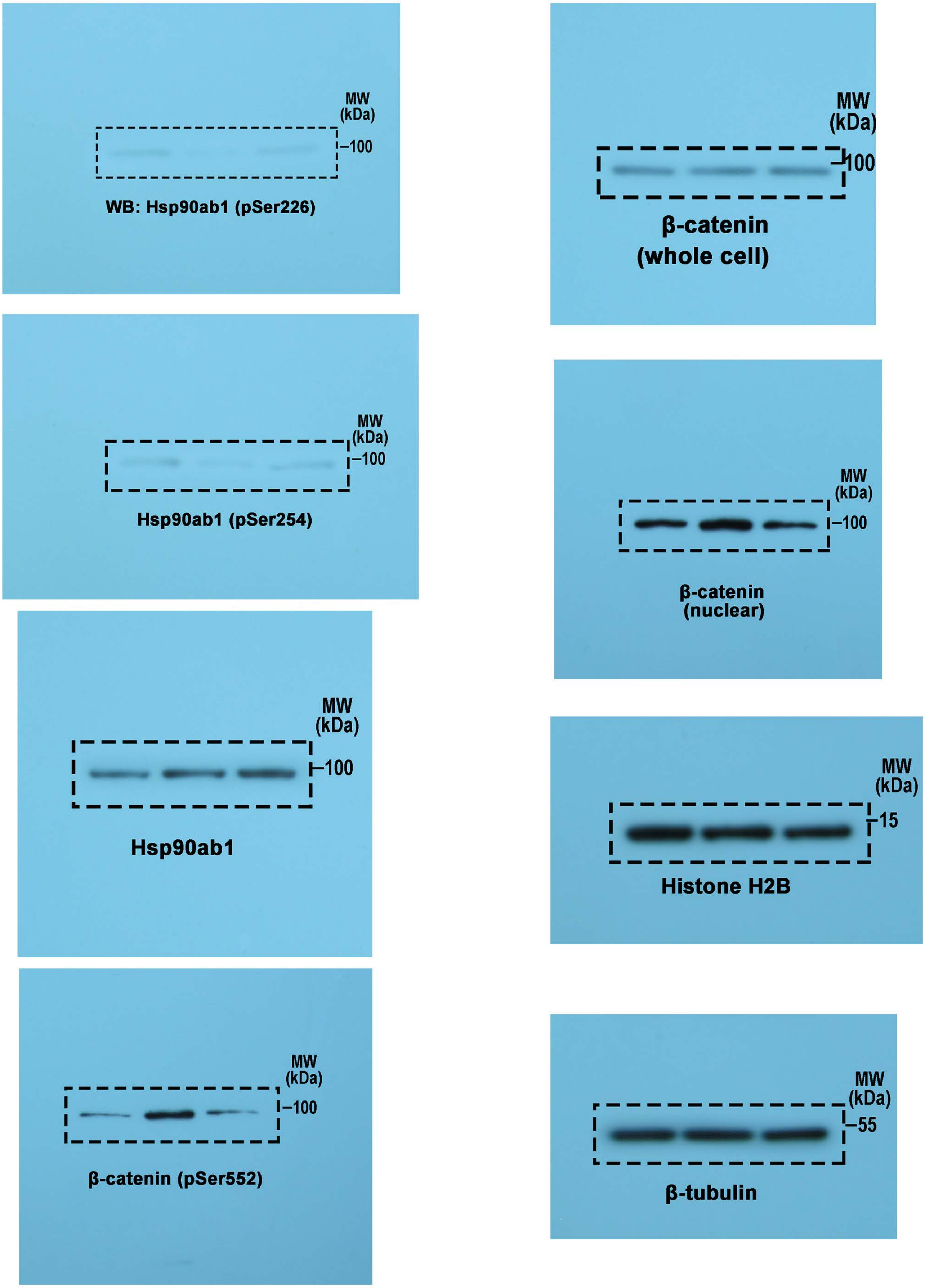

Fig 2e WB Full scan

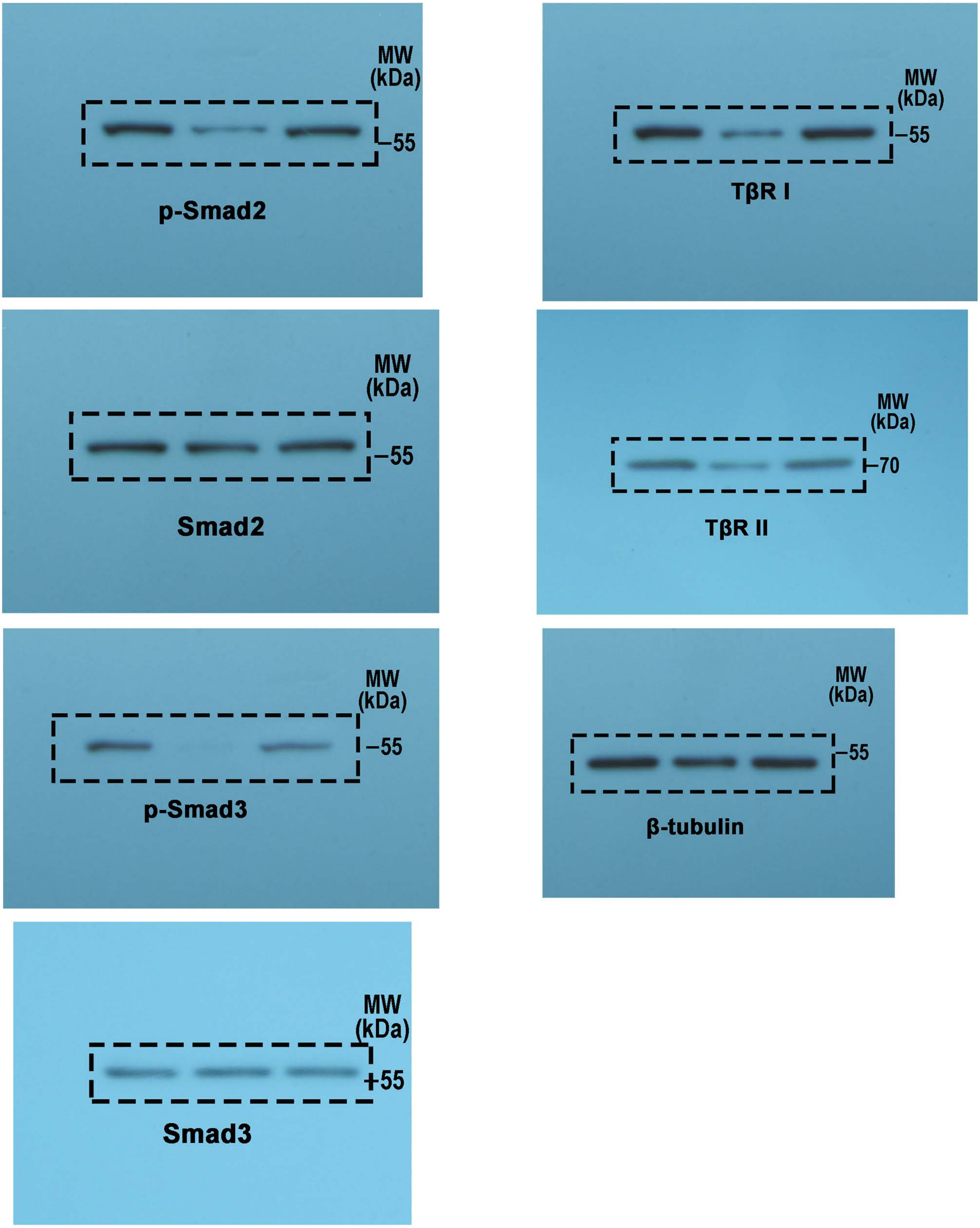

Fig 3a WB Full scan

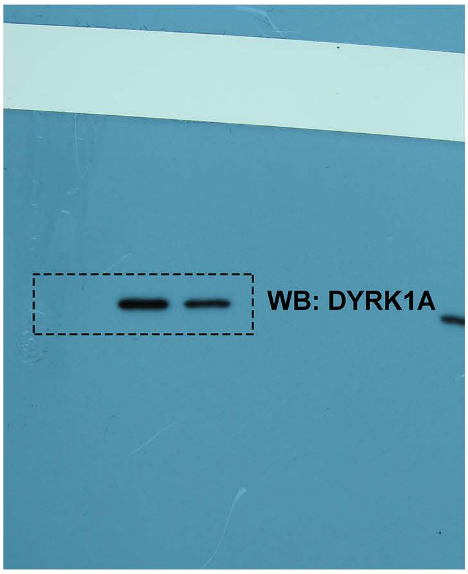

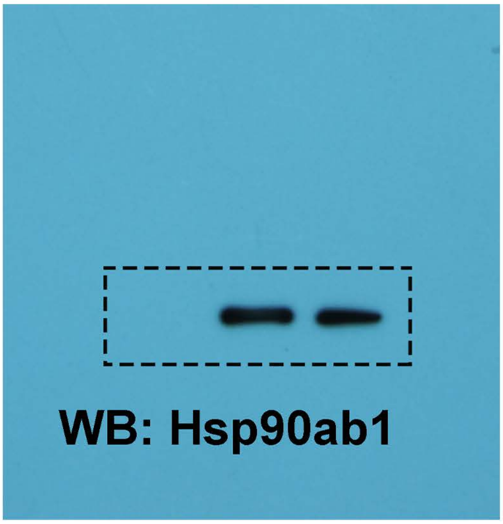

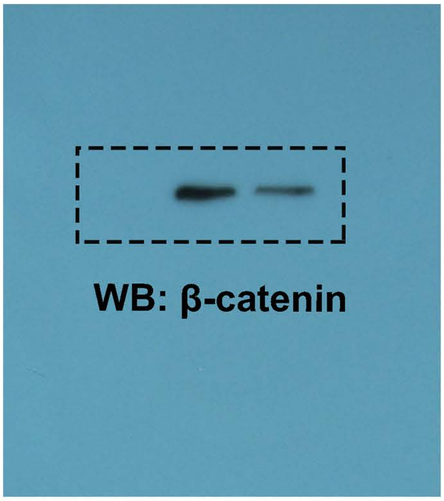

Fig 3c WB Full scan

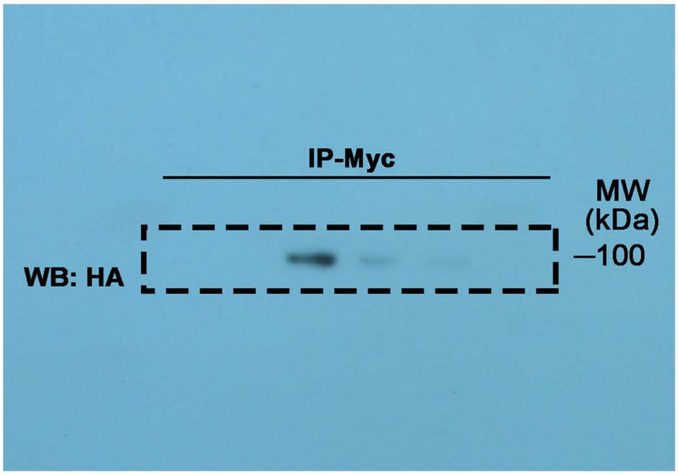

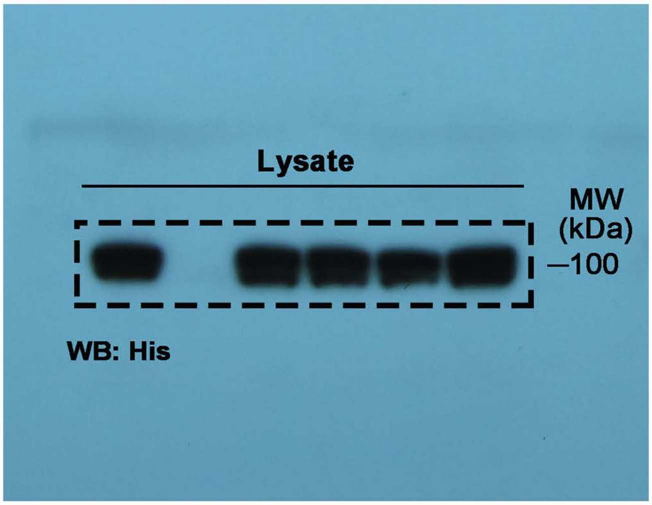

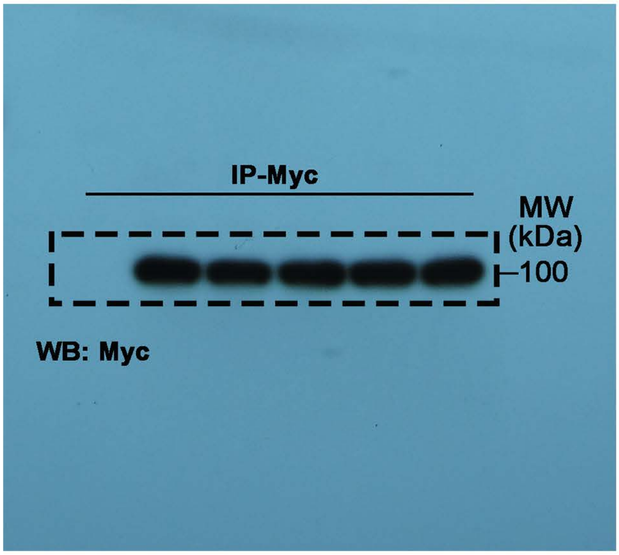

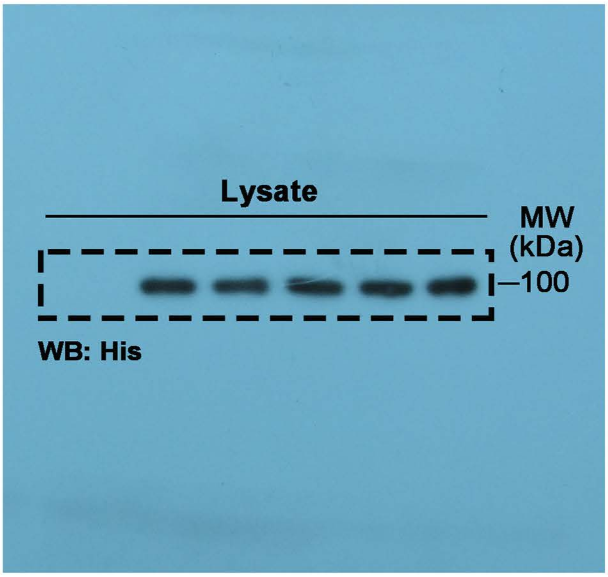

Fig 3e WB Full scan

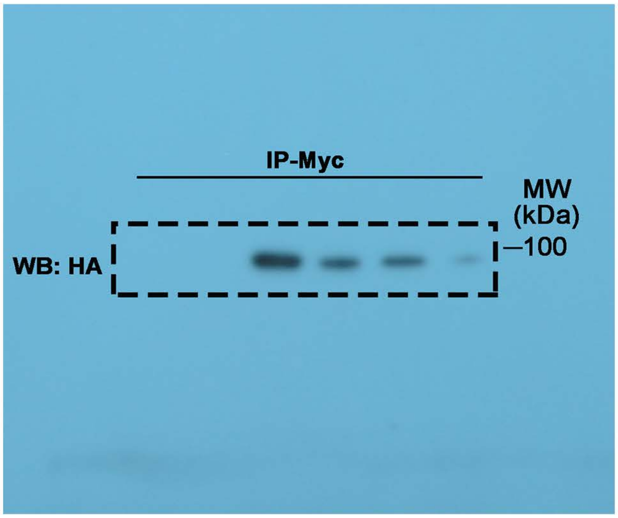

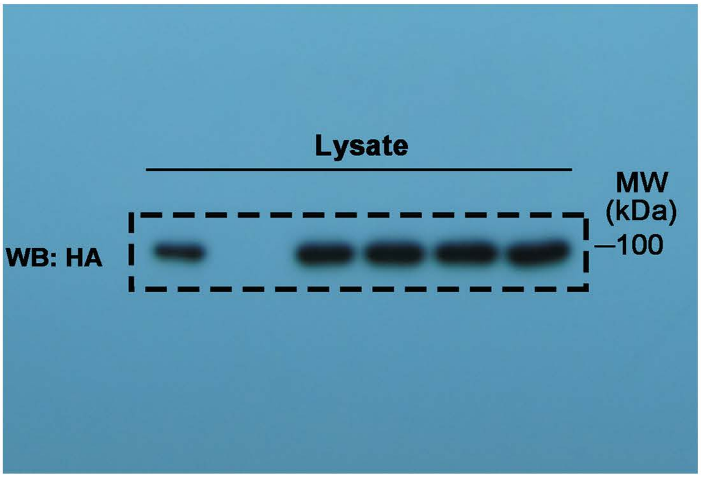

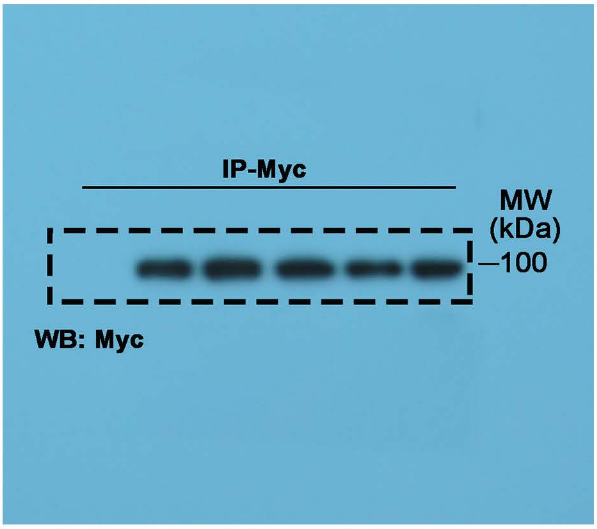

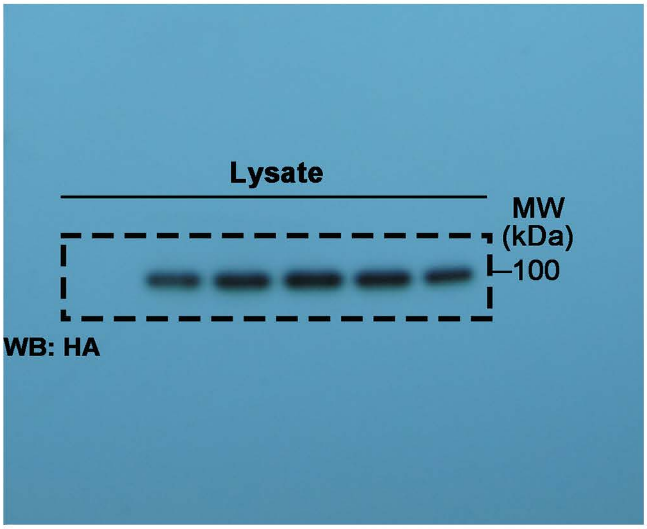

Fig 3g WB Full scan

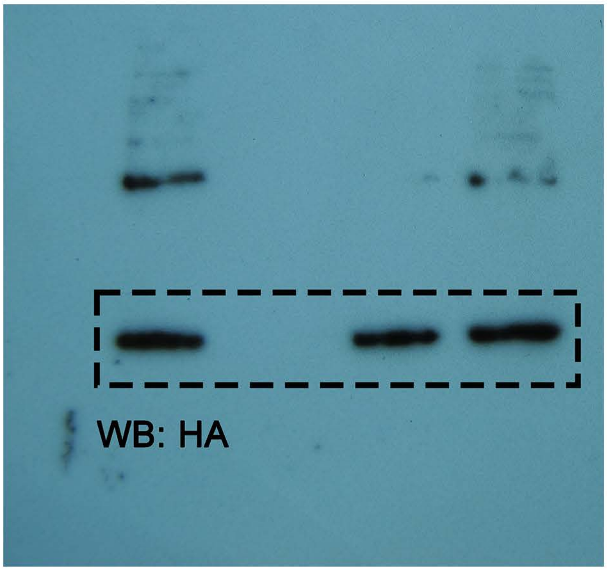

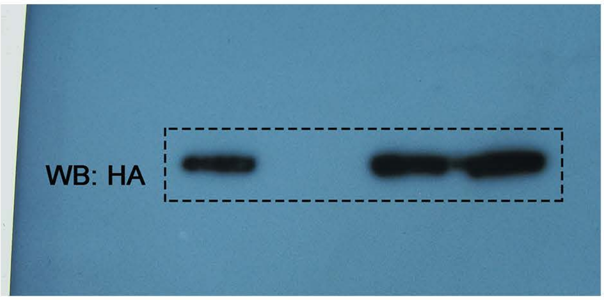

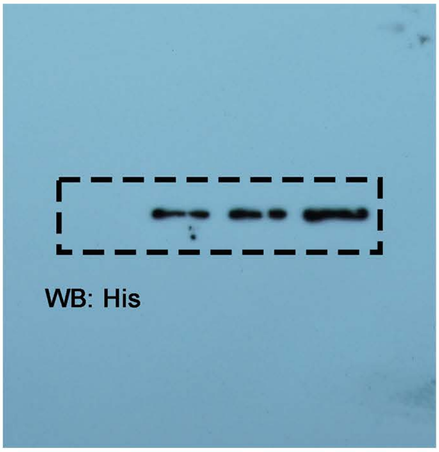

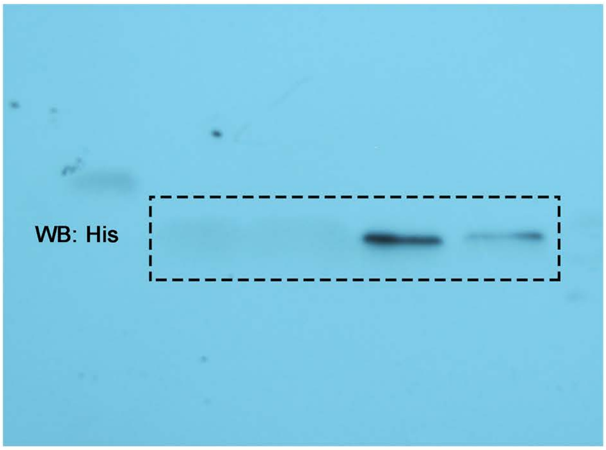

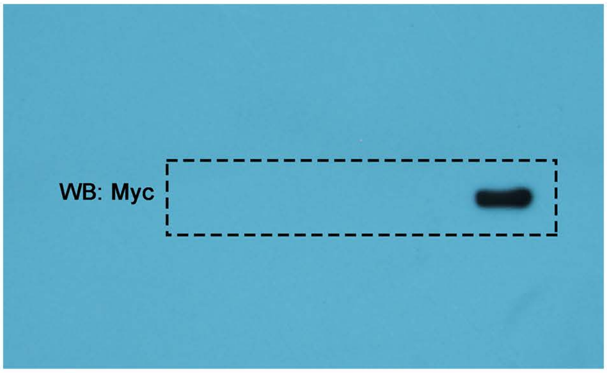

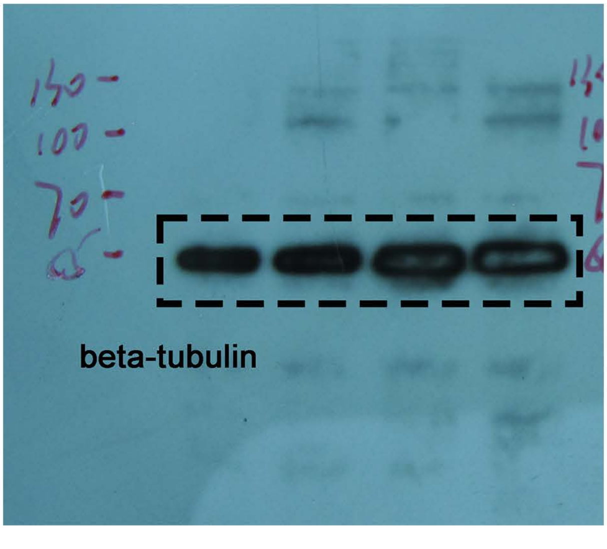

Fig 3h WB Full scan

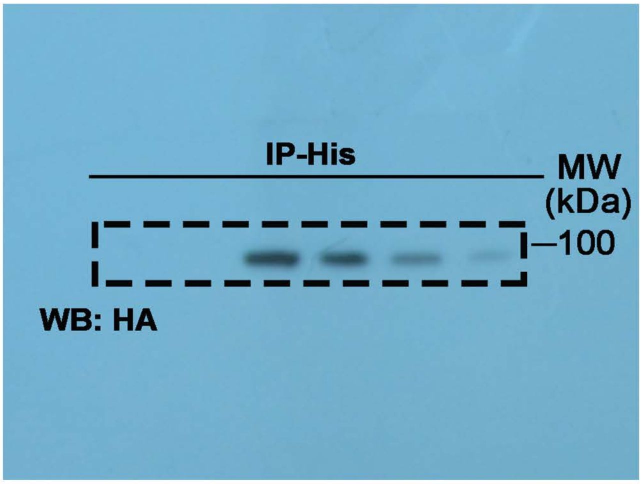

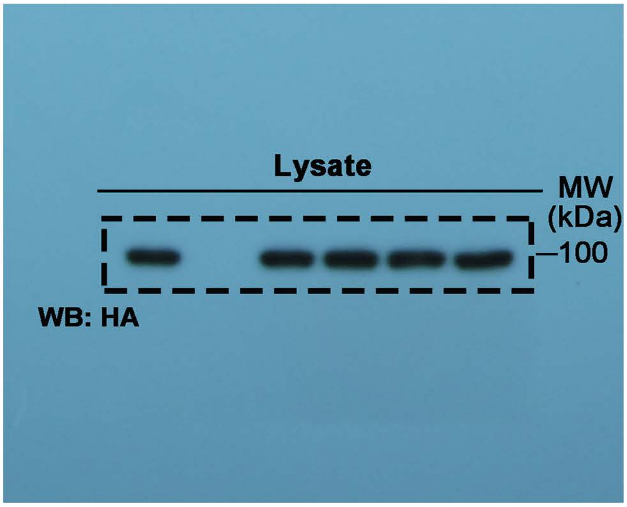

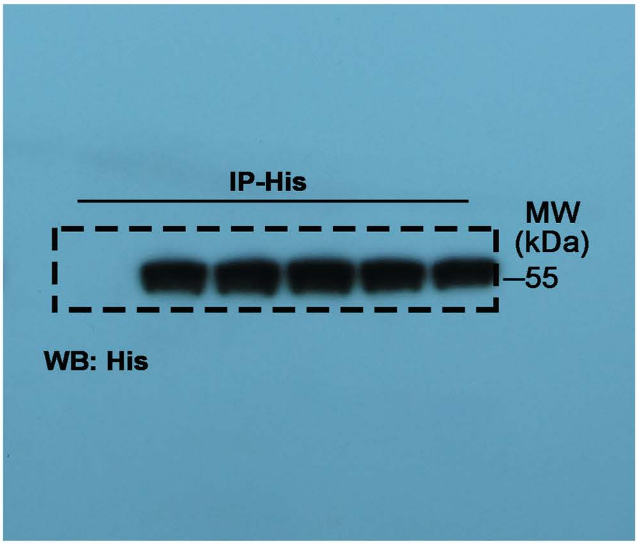

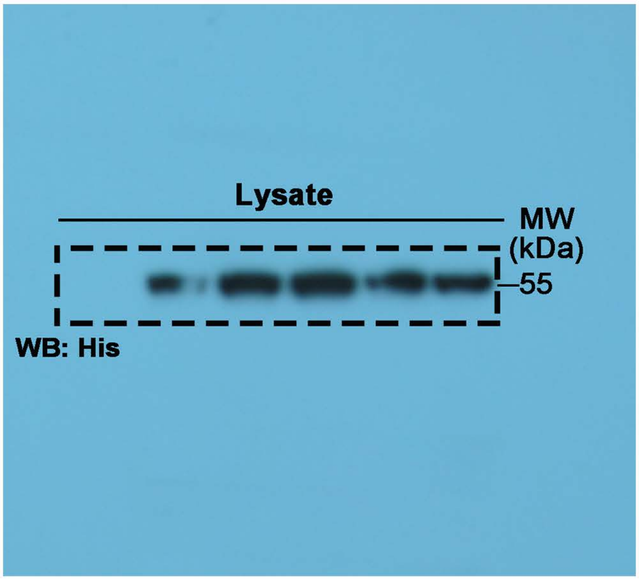

Fig 5a WB Full scan

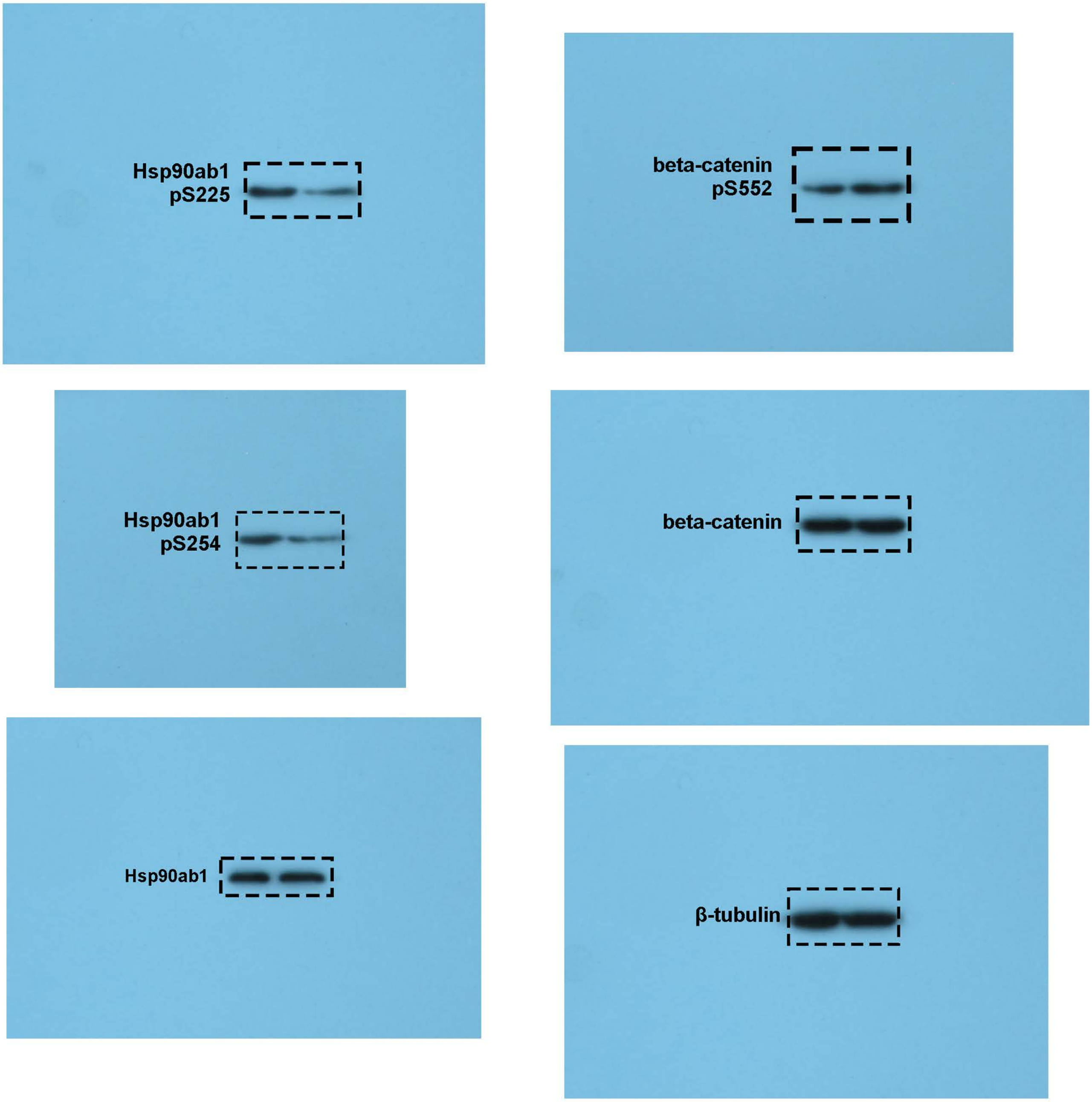

Extended Data Fig. 8a WB Full scan

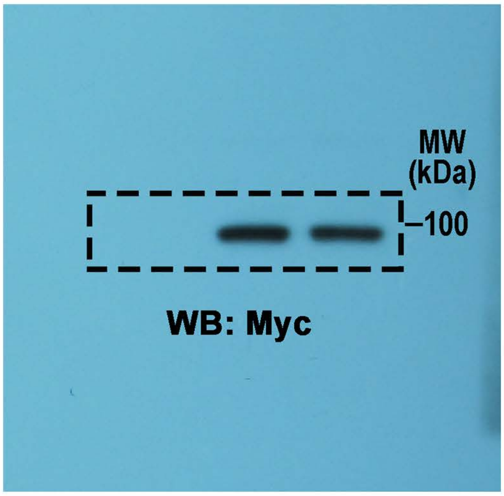

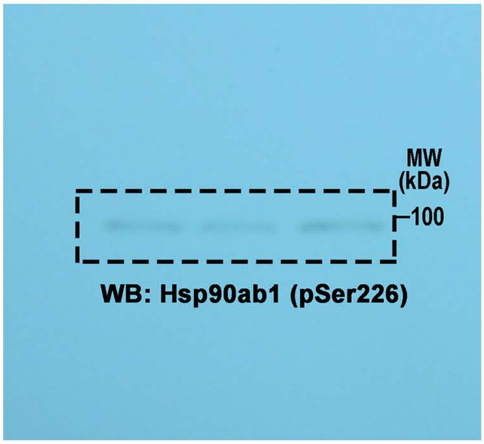

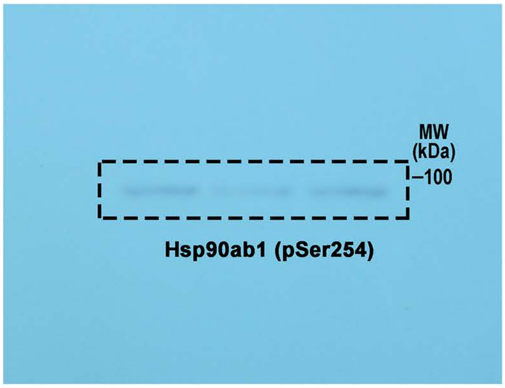

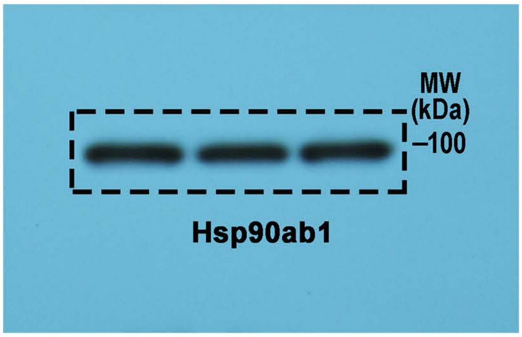

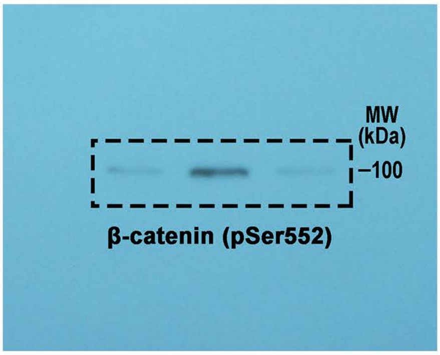

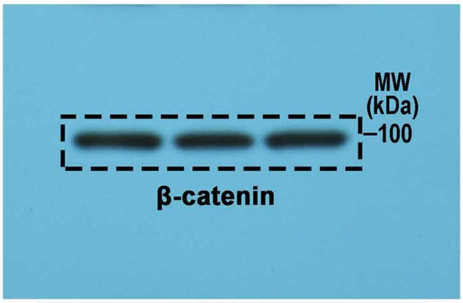

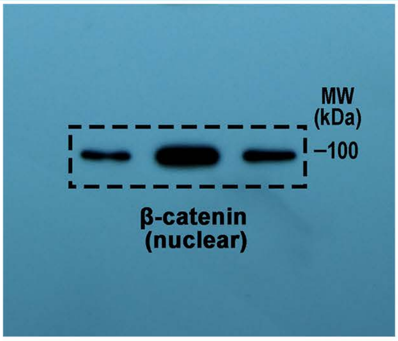

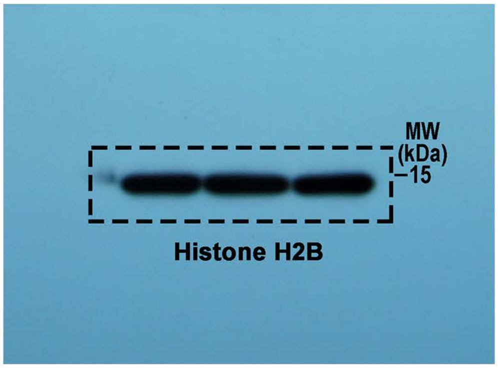

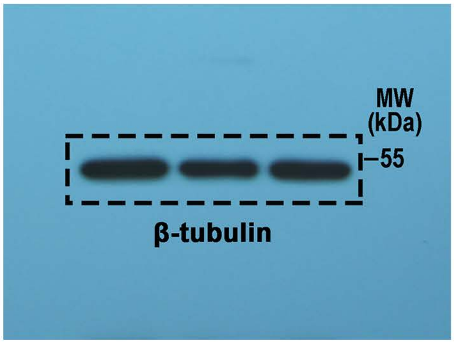

Extended Data Fig. 8b WB Full scan

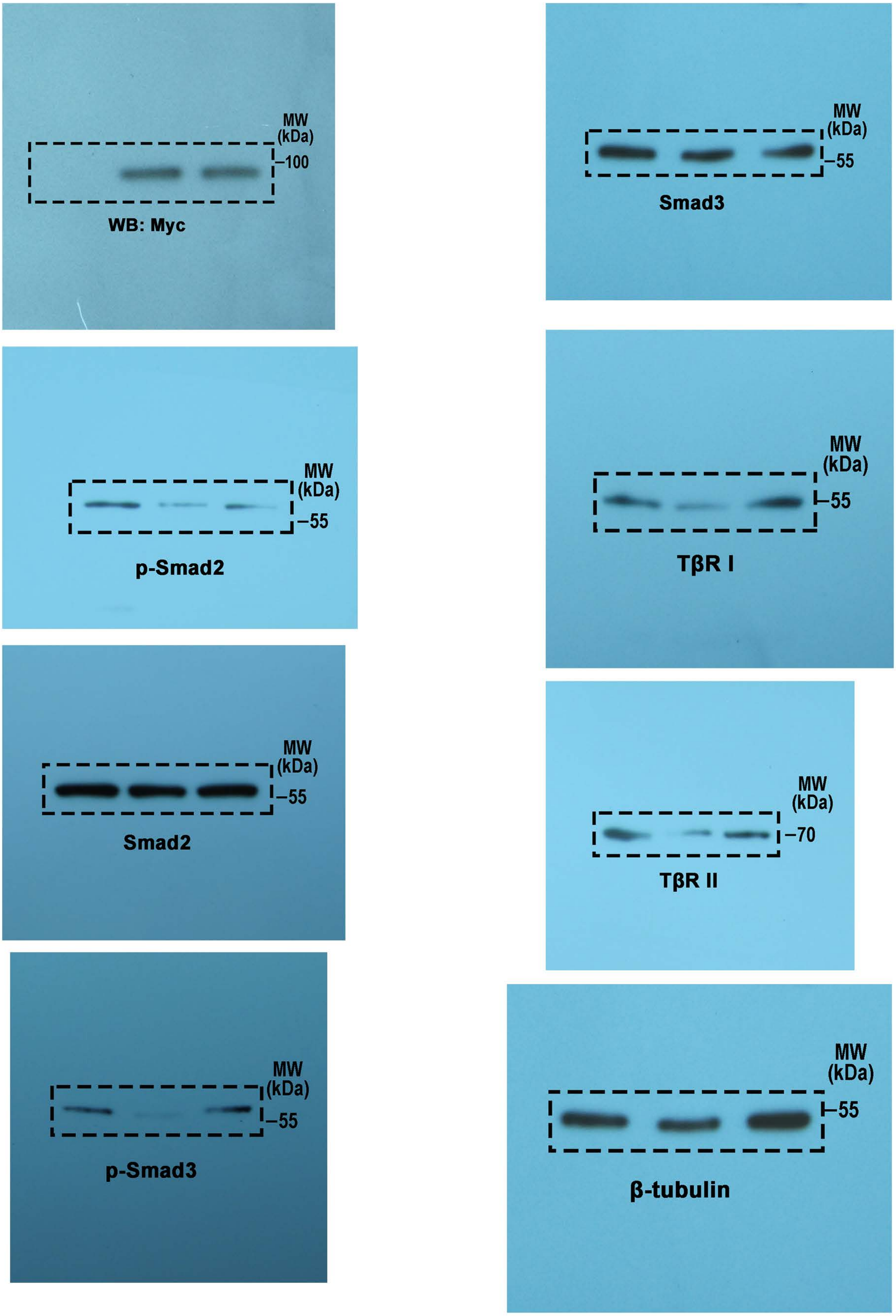

Extended Data Fig.11 WB Full scan

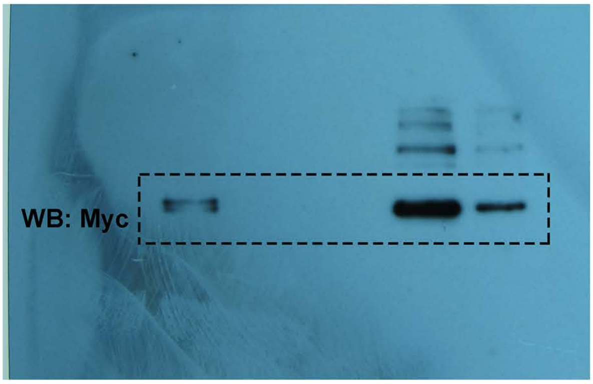

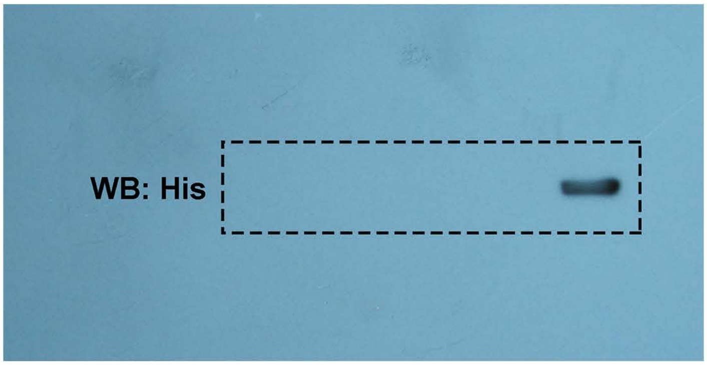

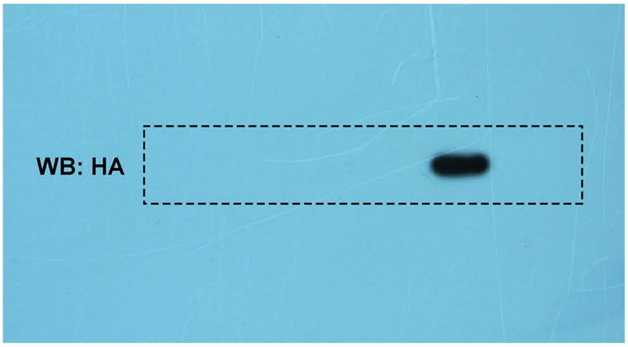

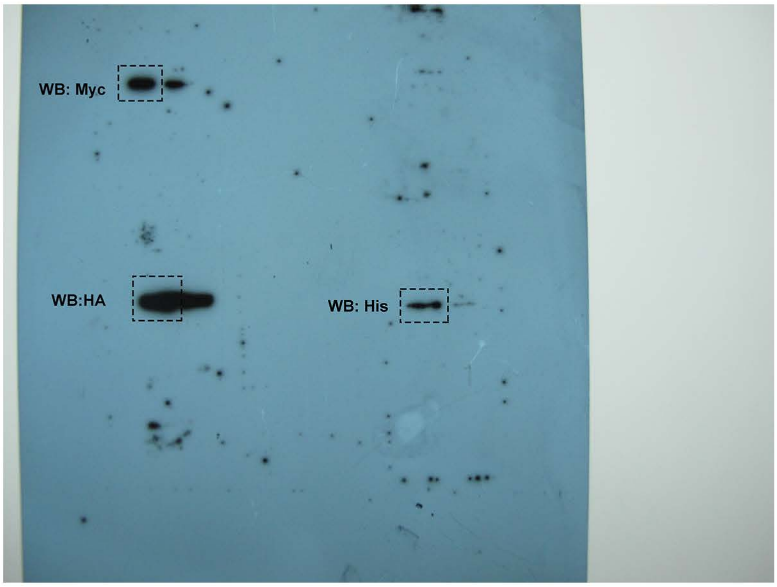

Extended Data Fig.13 WB Full scan

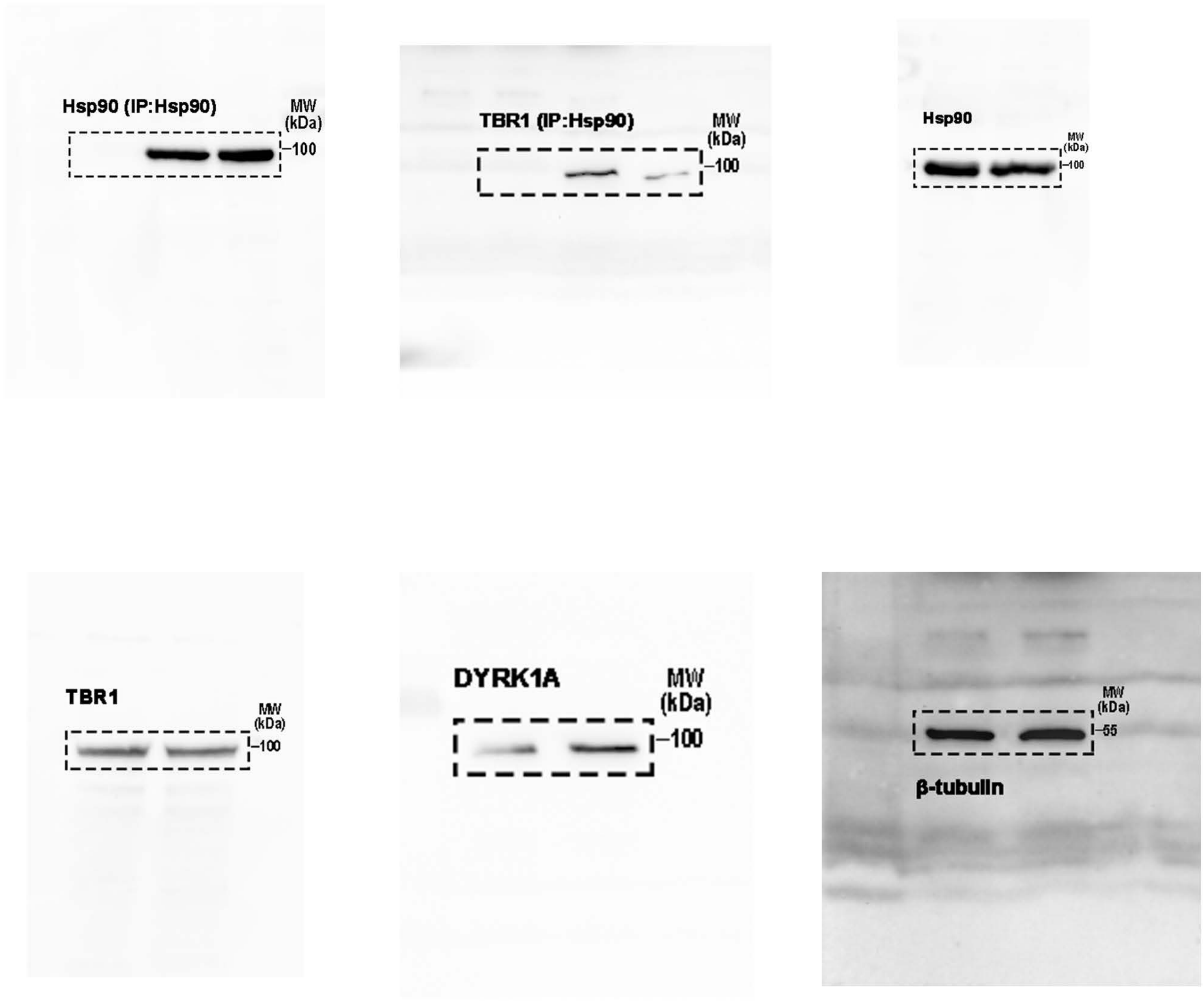

Extended Data Fig. 14 WB Full scan

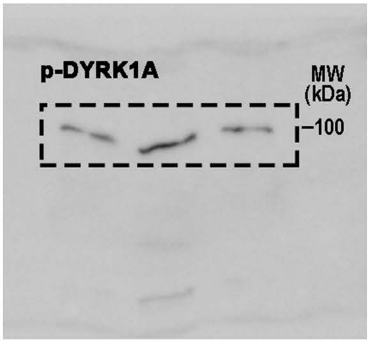

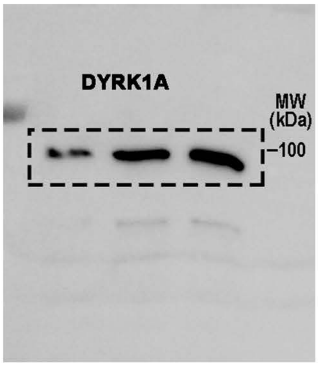

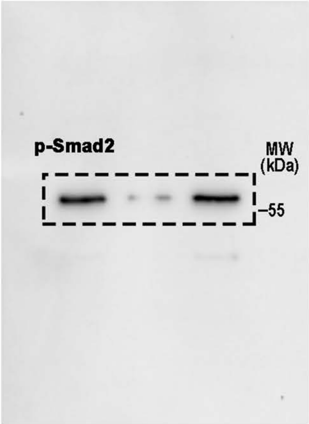

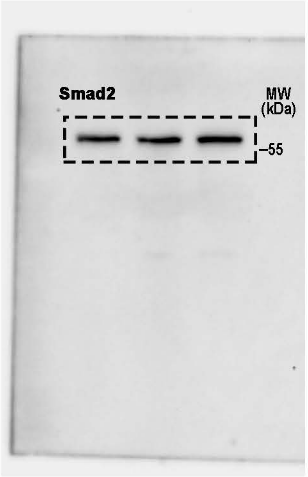

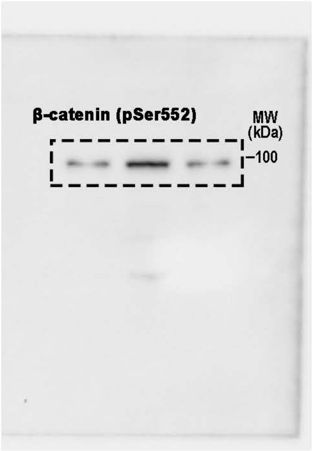

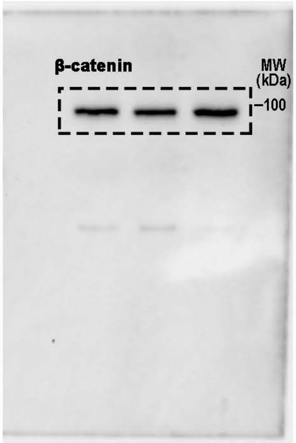

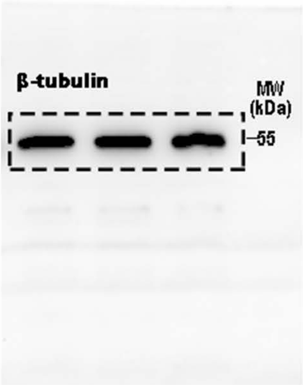

Supplement: Supplementary file 3 — original data files [file 41419_2022_5517_MOESM3_ESM.pdf]
